# Combining Multimodal Biomarkers to Guide Deep Brain Stimulation Programming in Parkinson Disease

**DOI:** 10.1016/j.neurom.2022.01.017

**Published:** 2022-02-23

**Authors:** Ashesh Shah, Thuy-Anh Khoa Nguyen, Katrin Peterman, Saed Khawaldeh, Ines Debove, Syed Ahmar Shah, Flavie Torrecillos, Huiling Tan, Alek Pogosyan, Martin Lenard Lachenmayer, Joan Michelis, Peter Brown, Claudio Pollo, Paul Krack, Andreas Nowacki, Gerd Tinkhauser

**Affiliations:** 1 Department of Neurology, Bern University Hospital, University of Bern, Bern, Switzerland; 2 Department of Neurosurgery, Bern University Hospital, University of Bern, Bern, Switzerland; 3 ARTORG Center for Biomedical Engineering Research, University of Bern, Bern, Switzerland; 4 MRC Brain Network Dynamics Unit, University of Oxford, Oxford, UK; 5 Nuffield Department of Clinical Neurosciences, University of Oxford, Oxford, UK; 6 Oxford Centre for Human Brain Activity, Wellcome Centre for Integrative Neuroimaging, Department of Psychiatry, University of Oxford, Oxford, UK; 7 Usher Institute, Edinburgh Medical School, The University of Edinburgh, Edinburgh, UK

**Keywords:** DBS programming, deep brain stimulation, local field potentials, Parkinson disease, subthalamic nucleus

## Abstract

**Background:**

Deep brain stimulation (DBS) programming of multicontact DBS leads relies on a very time-consuming manual screening procedure, and strategies to speed up this process are needed. Beta activity in subthalamic nucleus (STN) local field potentials (LFP) has been suggested as a promising marker to index optimal stimulation contacts in patients with Parkinson disease.

**Objective:**

In this study, we investigate the advantage of algorithmic selection and combination of multiple resting and movement state features from STN LFPs and imaging markers to predict three relevant clinical DBS parameters (clinical efficacy, therapeutic window, side-effect threshold).

**Materials and Methods:**

STN LFPs were recorded at rest and during voluntary movements from multicontact DBS leads in 27 hemispheres. Resting- and movement-state features from multiple frequency bands (alpha, low beta, high beta, gamma, fast gamma, high frequency oscillations [HFO]) were used to predict the clinical outcome parameters. Subanalyses included an anatomical stimulation sweet spot as an additional feature.

**Results:**

Both resting- and movement-state features contributed to the prediction, with resting (fast) gamma activity, resting/ movement-modulated beta activity, and movement-modulated HFO being most predictive. With the proposed algorithm, the best stimulation contact for the three clinical outcome parameters can be identified with a probability of almost 90% after considering half of the DBS lead contacts, and it outperforms the use of beta activity as single marker. The combination of electrophysiological and imaging markers can further improve the prediction.

**Conclusion:**

LFP-guided DBS programming based on algorithmic selection and combination of multiple electrophysiological and imaging markers can be an efficient approach to improve the clinical routine and outcome of DBS patients.

## Introduction

Detailed testing of deep brain stimulation (DBS) electrodes is an essential step for maximizing the outcome of DBS.^1,2^ However, this contact review is time-consuming, requires highly trained human resources, and is often exhausting for the patients who have to endure numerous evaluations.^3^ This limitation is even more evident for multicontact segmented DBS leads,^4,5^ which, because of the increased number of contacts and myriad of possible contact combinations, cannot be fully exploited using the current clinical approach.^6,7^ More objective, smart, and data-driven programming strategies are required to improve this aspect of DBS therapy.

A promising approach to inform DBS programming is by taking advantage of electrophysiological recordings from the implanted DBS lead. There is a large body of literature linking Parkinson disease (PD) symptoms to spectral features in basal ganglia signals, with exaggerated beta activity (13–30 Hz) being the best characterized. The latter is suggested to exhibit a limiting effect on the information coding capacity within the motor circuitry^8^ and thereby provoking bradykinesia and rigidity symptoms.^9–11^ Previous work by us and other groups demonstrated feasibility in using subthalamic nucleus (STN) beta activity at rest to inform DBS programming for ring contacts^12,13^ and segmented contacts.^14–16^ However, there are other potentially informative features, such as movement-related desynchronization of beta activity, which is associated with improved motor performance and localizes to the motor STN.^17–20^Gamma activity (60–100 Hz) synchronizes with movement, is viewed as prokinetic signal, and is also evident in the dorsal STN.^21–23^ High frequency oscillations (HFO) are similarly modulated as gamma activity, with the modulation being negatively correlated to brady-kinesia scores.^24,25^ In contrast, lower frequencies such as alpha activity (8–12 Hz) are more prevalent in the ventral STN^13^ and have been associated with nonmotor features.^26–28^

Overall, multiple spectral features at rest and movement as well as imaging markers could all be putative indicators of the optimal stimulation site. Following this, the current work aims to illustrate the benefit of an algorithmic selection and combination of multiple features to predict three relevant clinical DBS measurements (clinical efficacy, therapeutic window, and side-effect threshold) to optimize DBS programming.

## Materials and Methods

### Patients and Surgery

**Figure 1:**
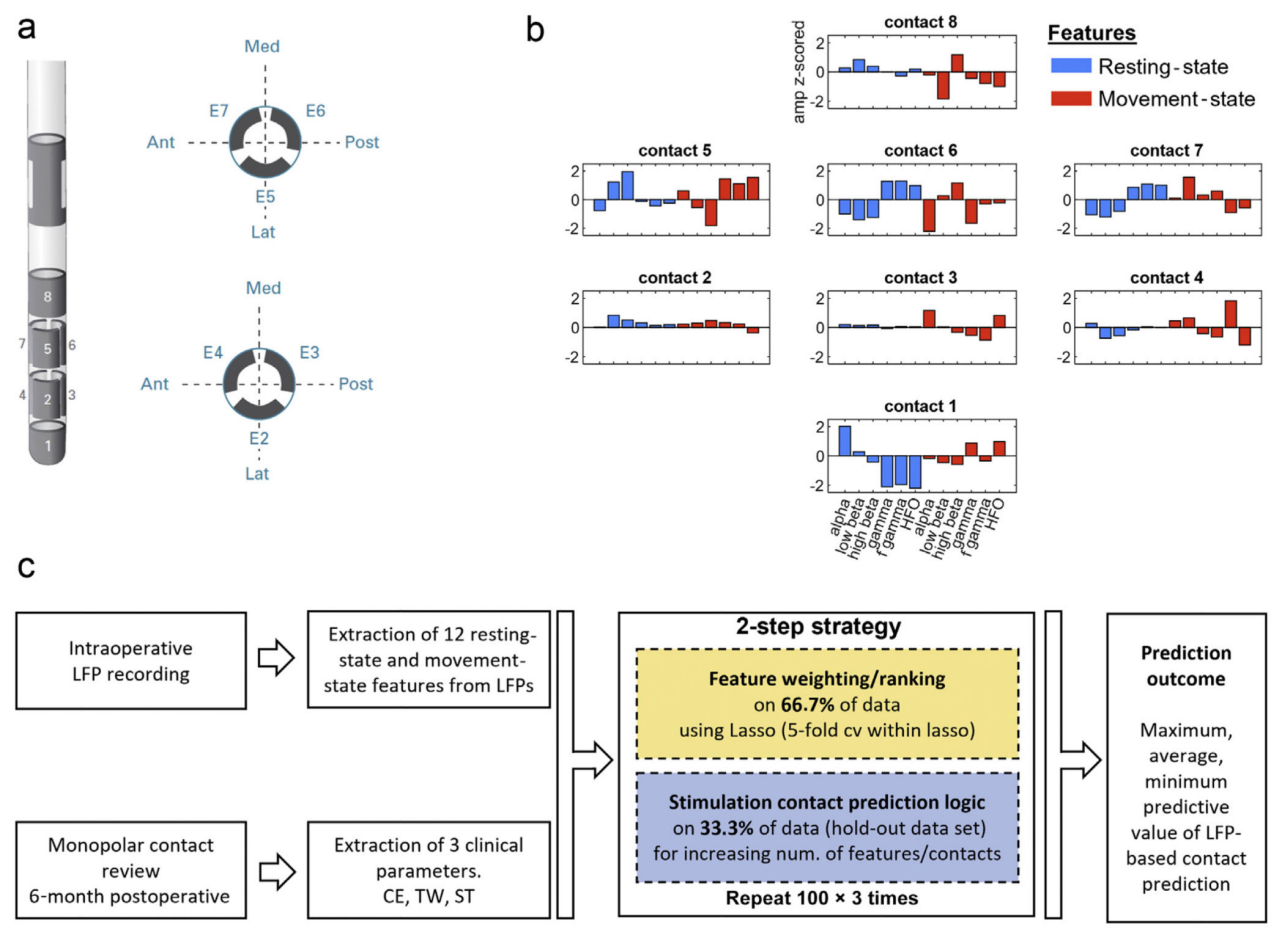
Method figure. a. Illustrates the directional DBS lead (Boston Scientific). Contacts are distributed along four levels. On levels two and three, there are three segmented contacts (level two: contacts 2/3/4; level three: contacts 5/6/7). b. For each contact, the resting state power- and movement-related modulation was derived for the following frequency bands: alpha (8–12 Hz), low beta (13–20 Hz), high beta (21–30 Hz), gamma (60–90 Hz), fast gamma (105–145 Hz), HFO (205–395 Hz). Hemisphere number 20 was selected as example to illustrate the heterogenous distribution of the features across the eight stimulation contacts (z-scoring of the amplitude performed separately for each feature category across the eight contacts). c. Flow chart summarizing the two-step strategy to validate the contact prediction performance. Ant, anterior; Med, medial; Post, posterior; Lat, lateral. [Color figure can be viewed at www.neuromodulationjournal.org]

In this study, we screened consecutive PD patients who under-went awake DBS surgery at the University Hospital in Bern from September 2017 to September 2019. The inclusion criteria were the following: 1) signed general consent (study approved by local ethics committee, 2017-00551); 2) local field potentials (LFPs) recorded at rest and during repetitive upper limb movements; 3) available systematic postoperative monopolar contact review of all stimulation contacts; and 4) two or more points in the contralateral upper limb rigidity score at baseline (off medication, off stimulation) during the postoperative clinical evaluation. After applying these criteria, a total of 27 hemispheres from 17 PD patients (11 male, 6 female), with a mean age at surgery of 58.6 ± 3.1 years and disease duration of 9.2 ± 1.2 years, were included (details in Supplementary Data Table S1). Patients were implanted with the Boston Vercise Cartesia directional leads (Boston Scientific, Marl-borough, MA) (Fig. 1a).

### Local Field Potential Recording and Intraoperative Assessment

LFPs were recorded during awake DBS surgery from all eight contacts simultaneously after the lead was placed in its final position and fixed to the skull. Recordings were performed with a TMSi-Porti amplifier (Twente Medical Systems International, Oldenzaal, The Netherlands), using a sampling frequency of 2048 Hz and common average referencing. In a first step, we performed LFP recording at rest (mean duration: 101.3 seconds ± 6.4 seconds). In a second step, patients were asked to perform contralateral upper limb movements that consisted of hand closing and opening. Surface electromyography electrodes and accelerometers were placed on the upper limb to objectively detect the movement onset. Each single movement was prompted by a verbal "go" command recorded with a microphone, and each movement was separated by an interval of 7.5 seconds ± 0.25 seconds (range: 6.1-11.3 seconds). This was to ensure that the movement-related rebound did not compromise the following trial.^29^ We aimed to record 20 such movement trials; the precise number was allowed to vary because of the intra-operative setting and associated constraints.

### Signal Processing

The raw signal of both rest and movement recordings was down-sampled to 1024 Hz and high-pass filtered at 5 Hz, and notch filters were set at 50 Hz and its harmonics (up to 400 Hz). For hemisphere 10, one directional contact and for hemisphere 22, one directional and one ring contact had to be excluded from the analysis because of signal saturation. To avoid biasing the feature selection (indicated subsequently in the text) because of potential differences in recorded voltage following different biophysical properties of the contacts or variability in impedances and hence, to maintain a comparable spectral distribution, the raw data was z-scored for each contact separately. Following this, the frequency decomposition was performed at 1 Hz resolution using the Wavelet method (ft_specest_wavelet script in FieldTrip—Morlet Wavelet, width = 12, gwidth = 5; Donders Institute for Brain, Cognition and Behaviour, 2010).

For the movement task, the continuous signal was segmented into four-second blocks centered around the movement onset. Trials containing signal artifacts (ie, owing to cable movement) were removed, leaving an average number of 17.4 ± 0.7 (range: 9–22) per hemisphere. The movement-related power modulation was estimated by normalizing the period from movement onset to 300 milliseconds after movement of each trial with respect to the baseline power averaged in a precue window ranging from –2 seconds to –1.5 seconds before movement and across trials. The movement-related modulation, which showed an event-related desynchronization (ERD) around the beta band and an event-related synchronization (ERS) around the gamma band, was then averaged across trials. For both resting and movement state, the following frequency bands were extracted for further analyses: alpha (8:12 Hz), low beta (13:20 Hz), high beta (21:30 Hz), gamma (60:90 Hz), fast gamma (105:145 Hz), and HFO (205:395 Hz). Figure 1b depicts an example with normalized features, and Supplementary Data Figure S1 illustrates the resting and movement spectral curves of the 12 electrophysiological features used.

### Spatial-Electrophysiological Processing

In subanalyses, we reevaluated the contact prediction performance after adding an anatomical landmark to the feature list. As anatomical landmark feature, we set the minimum distance (Euclidean distance) of the contact center to a previously published volume of tissue activated (VTA)-based anatomical reference point (stimulation sweet spot indicating a low effect threshold) within the normalized space of the motor STN (Montreal Neurological Institute x: 12.5838 y: –12.4868 z: –6.2879).^30^ Lead reconstruction was performed with the Lead-DBS toolbox^31,32^ (details in the Supplementary Material).

### Postoperative Clinical Assessment and Clinical Outcome Parameter

The postoperative monopolar contact review served as the main clinical outcome parameter to determine the value of LFPs to predict the optimal stimulation contacts. This assessment took place after 5.4 months (range 4–10 months) postoperatively to avoid major DBS stun effects^33^ and was performed by the DBS-team, blinded to LFP data. Clinical contact testing of each segmented ring level and all directional contacts followed the standardized monopolar contact review procedures^2^ after dopaminergic medication was withdrawn at least 12 hours and dopamine agonists up to 48 hours before testing. Clinical assessment focused on upper limb rigidity testing. First, the effect threshold (ET) was determined as the stimulation current necessary to completely relieve rigidity or to obtain the best achievable improvement. The side-effect threshold (ST) was defined as the stimulation current where limiting side effects occurred. Both ET and ST were determined by 0.5 mA incremental steps. Stimulation frequency and pulse width were set to 130 Hz and 60 μs by default. Therapeutic window (TW) was defined as the difference between ST and ET. DBS clinical efficacy was defined according to the following formula:

$$Clinical\;efficacy=\frac{100\times \left(rigidity\;at\;baseline\;-rigitidy\;at\;ET\right)}{rigitidy\;at\;baseline\times Current\;at\;ET}$$

Only hemispheres with at least two points in Movement Disorder Society Unified Parkinson's Disease Rating Scale–assessed upper limb rigidity at baseline (off stimulation/medication) were used, to increase the dynamic range of the clinical response.

In addition to the postoperative clinical assessment at 5.4 months, we assessed the stimulation setting used at around one year after operation (12.5 months, range 11–15 months) for further comparisons.

### Features Selection, Contact Prediction, and Statistical Analyses

All statistical analyses were performed using MATLAB (2019b; Mathworks, Natick, MA). To study the contact prediction accuracy, a two-step contact prediction method was implemented.

Step one includes the weighting and ranking of 12 electro-physiological features based on a Lasso regression with fivefold cross-validation performed on 66.7% of the data set (18/27 hemispheres). Step two uses previously developed prediction logic to predict cumulative probability of choosing the best contact on the remaining 33.3% of the data set (9/27 hemispheres). The inputs for step one (Lasso regression) are the 12 spectral features extracted from the LFP recordings as variables and the three clinical features (CE, TW, and ST) as the outcome measures, normalized (z-scored) within-feature category and hemisphere. The output of step one is a list of ranked spectral features and their corresponding weights to predict the best CE, TW, and ST.

Step two corresponds to a previously published prediction logic, where the contacts are ranked (from presumed best LFP-contact to worst LFP-contact) using the weights of five of the best spectral features (output of step one). The algorithm iterates through the LFP-based contact ranking and determines how many contacts need to be tested before the one with the best clinical metric (CE, TW, or ST) is identified. This is done individually for nine of the 27 hemispheres. The output of this stage is the relative number of hemispheres (in percent) for the incrementing number of contacts to be tested (from one to maximum) to determine the best clinical contact. This is termed as "likelihood" of predicting the optimal stimulation contact.

To increase the generalizability, this two-step method was repeated for 100 iterations, with random allocation of the hemispheres during each iteration to either the training (66.7%) data set or testing (33.3%, hold-out) data set. Consequently, we report the maximum, average, and minimum likelihoods of predicting the optimal stimulation contact using different feature combinations over these iterations. We would like to highlight that the over-arching goal of this work was to identify the optimal simulation contact and not to predict the clinical metric values (as a continuous measure). The pipeline is illustrated in Figure 1c and further detailed in the Supplementary Material section.

It should be noted that this two-step method was performed twice independently, once for the six centered segmented contacts only (referred to in the text as "segmented contacts") as input and once for all eight contacts (six segmented contacts + two ring contacts, referred to in the text as "all contacts") as input. Correspondingly, two sets of results are described for 1) segmented contacts and 2) all contacts. This distinction was made because segmented and ring contacts might differ in their biophysical properties, location, spatial resolution, and clinical response to stimulation. Given that the implantation strategy of DBS electrodes, as well as the programming strategy in terms of omnidirectional/directional stimulation, might vary from one to another DBS center, we are presenting the contact prediction accuracy for both electrode subsets.

Statistical tests used are explicitly outlined (Friedmann test for repeated measurements and one-sampled *t*-tests for single comparison). *p* Values were corrected for multiple comparisons using false discovery rate.

## Results

### Spectral Features and Clinical Relationship

**Figure 2:**
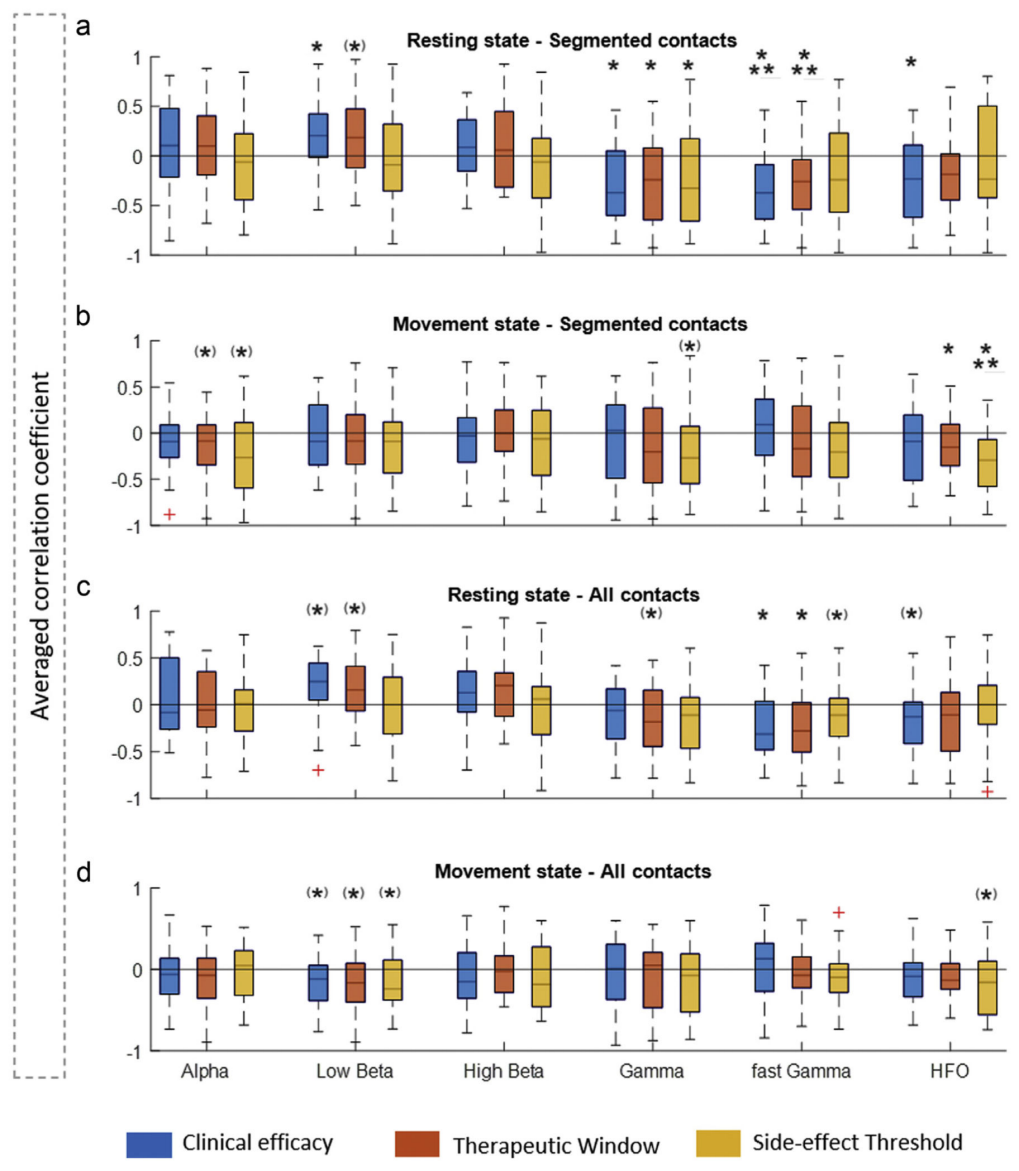
Clinical and spectral relationship. Shows the averaged r-values from Spearman's correlations performed for every hemisphere (n = 27) between the clinical DBS parameters (CE in blue, TW in red, ST in yellow) and the electrophysiological features (alpha 8–12 Hz, low beta 13–20 Hz, high beta 13–30 Hz, gamma 60–90 Hz, fast gamma 105–145 Hz, and HFO 205–395 Hz) separately for the resting state/segmented contacts (a), movement state/segmented contacts (b), resting state/all contacts (c), and movement state/all contacts (d). For all different sets of r-values across hemisphere, a one-sampled *t*-test was performed to illustrate the consistency of the trends of the correlation coefficients. Values are represented as mean + SEM. *p < 0.05; ***p < 0.001; (*)*p* value significant before correction for multiple comparisons (FDR) only. [Color figure can be viewed at www.neuromodulationjournal.org]

Figure 2 illustrates the bivariate correlative relationship within the DBS lead between the multiple LFP resting- and movement-state features with the three clinical outcome parameters (CE, TW, and ST) for DBS programming. This is intended to provide a visual illustration of the overall trend between single spectral features and clinical parameters. For segmented contacts, CE correlates positively with rest low beta (13:20 Hz) and negatively with rest gamma (60:90 Hz), fast gamma (105:145 Hz), and HFO (205:395 Hz). For all contacts, only the resting state fast gamma is negatively correlated with CE. Similarly, for TW, considering segmented contacts, we see a negative correlation with resting state gamma and fast gamma. Considering all contacts, TW has a negative correlation with resting state fast gamma. Finally, the ST for segmented contacts has a negative correlation with resting gamma and movement-related HFO modulation, whereas for all contacts, it is negatively correlated with resting gamma. Moreover, the within-feature correlation is weak, which mitigates against information redundancy of the features (Supplementary Data Fig. S2).

### Feature Selection and Ranking

**Figure 3:**
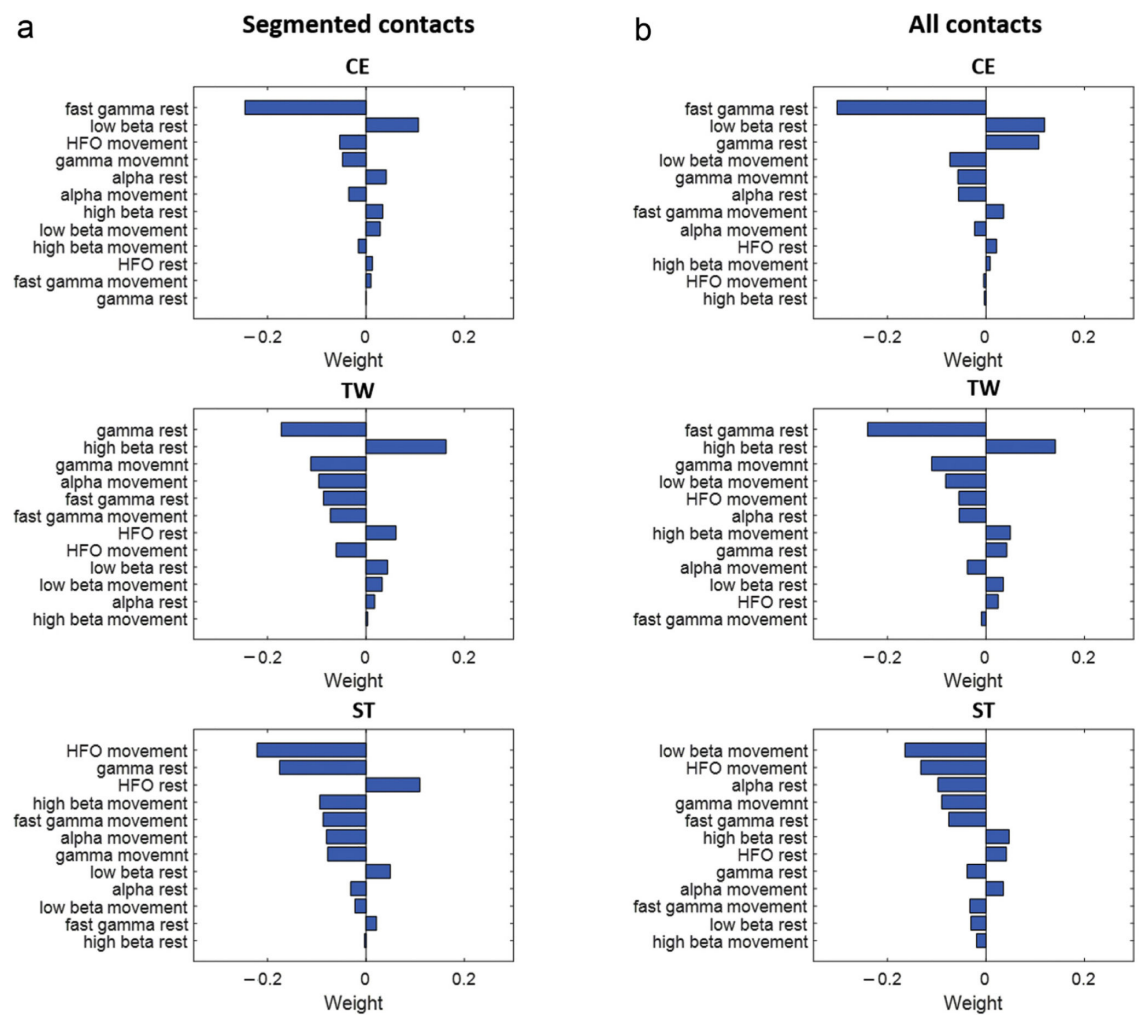
Feature ranking. Illustrates the output of step one of the two-step contact prediction method, corresponding to the ranked weights of the spectral features as determined by the Lasso regression. The features are ranked in descending order of predictive value for segmented contacts (a) and all contacts (b) and separately for the different clinical parameters (CE, TW, ST). The weights are averaged across the various iterations (Fig. 1c). The two most predictive features for each depicted configuration are the following: the most predictive feature for CE for both (a) and (b) is resting-state fast gamma activity (negative relationship), followed by resting-state low beta activity (positive relationship). For TW, the feature ranking results are similar, with the most predictive features being resting-state gamma activity (negative relationship) in (a) and resting-state fast gamma activity (negative relationship) in panel b. The second most predictive feature for both contact configurations is high beta activity (positive relationship). For the ST, the most predictive feature is movement-state HFO (negative relationship), followed by gamma at rest (negative relationship) in panel a. In panel b, the most predictive feature is low beta ERD (negative relationship), followed by movement-state HFO (negative relationship). [Color figure can be viewed at www.neuromodulationjournal.org]

In the previous section, we demonstrated the averaged correlative trend between features and clinical DBS outcome parameters. However, to select and weight the best spectral features for the contact prediction, we implemented Lasso regression in our analytical pipeline. This method takes all apparently weak predictors and assesses the correlations of each predictor with the outcome of interest in combination with all the other predictors, and accordingly assigns weights to each predictor. In addition, Lasso regression can simultaneously aid in feature selection by forcing unimportant features to have a coefficient of zero (effectively "knocking" them out of the trained model). The resulting feature, ranking from high to low predictive value and separately for the different clinical parameters, is illustrated in Figure 3a for segmented contacts and Figure 3b for all contacts. The most predictive feature for CE for both segmented contacts and all contacts is resting-state fast gamma activity (negative correlation), followed by resting-state low beta activity (positive correlation). Similarly, for TW and segmented contacts, the most predictive features are resting-state gamma activity (negative correlation), and considering all contacts, the most predictive feature is resting-state fast gamma activity (negative correlation). The second most predictive feature for both configurations is high beta activity (positive correlation). In contrast, the ST is best explained by movement-state HFO (negative correlation) and gamma at rest (negative correlation) for segmented contacts. Movement-related modulation of low beta and HFO (both negative correlation) is most predictive for the ST for all contacts. The consistency of these findings is supported by similar weights and correlative directions of the features found in the Lasso regression as well as in the simple bivariate-within hemisphere-correlations (Fig. 2).

### Contact Prediction Performance

**Figure 4:**
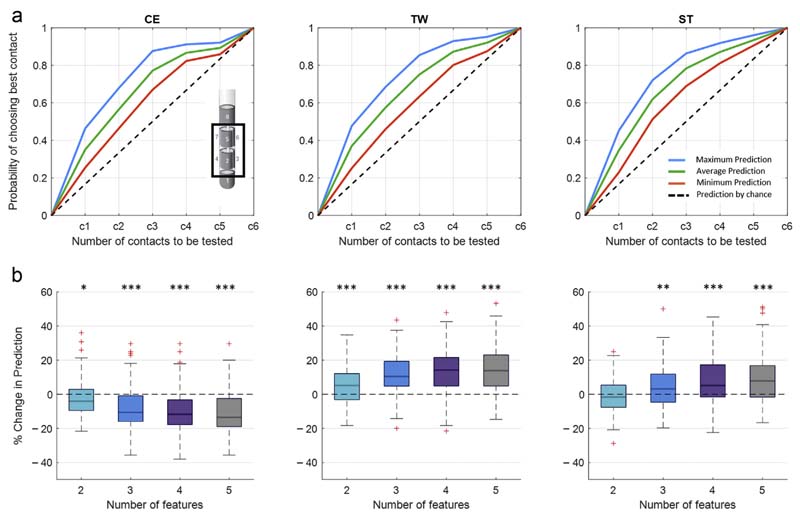
LFP-based contact prediction for segmented contacts. a. Illustrates the output of the second step of the contact prediction method, corresponding to the probability of identifying the best stimulation contact for the three clinical parameters (CE, TW, ST) out of the six segmented contacts. Within each subplot, the maximum, average, and minimum prediction accuracies evaluated on the hold-out set of nine hemispheres are illustrated as mean value, averaged across the multiple iteration of the prediction pipeline (Fig. 1). The dashed black lines illustrate the prediction by chance (conventional gold standard test strategy), where the probability of identifying the most efficient stimulation contact increases by 0.17 after each contact tested. By applying the LFP-based contact prediction strategy and considering half of the stimulation contacts, the probability of identifying the best stimulation contacts can reach a maximum as follows: CE: 88%, TW: 85%, and ST: 86%. These results are derived as overall output of the prediction algorithm, after combining the five best ranked features, without differentiating whether the best selected features or the stepwise combination of features more strongly predicts the optimal stimulation site. b. Illustrates the percentage change in contact prediction, considering half of the stimulation contacts (3/6), following the combination of multiple features relative to the use of the single highest ranked feature for the three clinical parameters (CE, TW, ST). For CE, adding additional features leads to a reduction of the prediction performance (Friedmann test: x^2^ (3) = 36.26, *p* ≤ 0.001; significant one-sampled *t*-test for number of two, three, four, five features). For TW (Friedmann test: x^2^ (3) = 36.88, *p* ≤ 0.001; sign. one-sampled *t*-test for number of two, three, four, five features) and ST (Friedmann test: x^2^ (3) = 28.94, *p* ≤ 0.001; significant one-sampled *t*-test for number of two, three, four, five features), the prediction performance can be increased by combining multiple features. p Values were false discovery rate corrected. *p < 0.05; **p < 0.01; ***p < 0.001. Detailed statistics in the Supplementary Material. [Color figure can be viewed at www.neuromodulationjournal.org]

**Figure 5:**
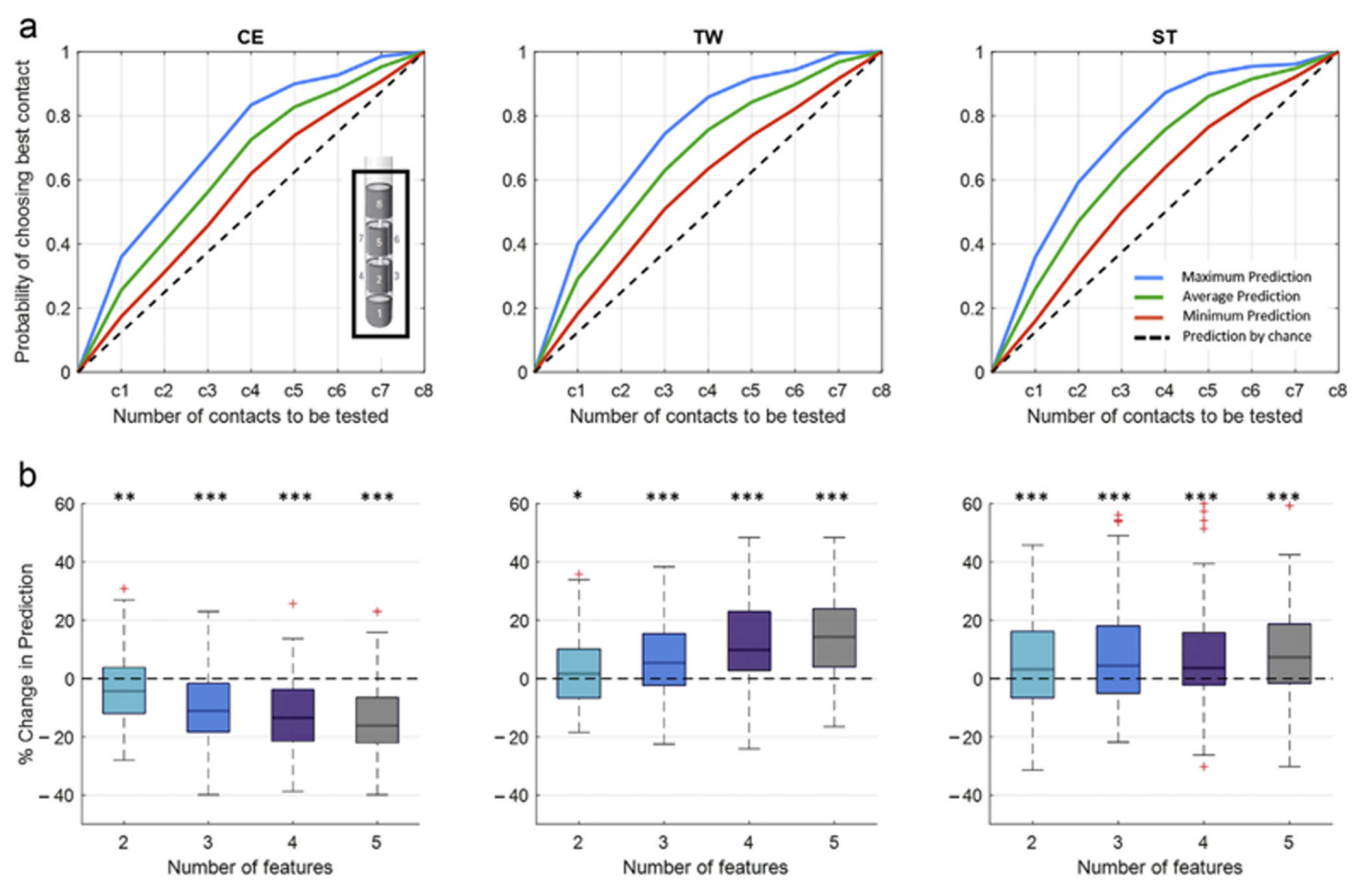
LFP-based contact prediction for all contacts. a. Illustrates the output of the second step of the contact prediction method, corresponding to the probability of identifying the best stimulation contact for the three clinical parameters (CE,TW, ST) out of the eight contacts across. Within each subplot, the maximum, average, and minimum prediction accuracies evaluated on the hold-outset of nine hemispheres are illustrated as mean value, averaged across the multiple iteration of the prediction pipeline (Fig. 1). The dashed black lines illustrate the prediction by chance (conventional gold standard test strategy), where the probability of identifying the most efficient stimulation contact increases by 0.125 after each contact tested. By applying the LFP-based contact prediction strategy and considering half of the stimulation contacts, the probability of identifying the best stimulation contacts can reach a maximum as follows: CE: 83%, TW: 86%, and ST: 87%. These results are derived as overall output of the prediction algorithm, after combining the five best ranked features, without differentiating whether the best selected features or the stepwise combination of features more strongly predicts the optimal stimulation site. b. Illustrates the percentage change in contact prediction, considering half of the stimulation contacts (4/ 8), following the combination of multiple features relative to the use of the single highest ranked feature for the three clinical parameters (CE, TW, ST). For CE, adding additional features leads to a reduction of the prediction performance (Friedmann test: x^2^ (3) = 53.94, *p* ≤ 0.001; significant one-sampled *t*-test for number of two, three, four, five features). For TW (Friedmann test: x (3) = 50.25, *p* ≤ 0.001; significant one-sampled *t*-test for number of two, three, four, five features), the prediction performance can be increased by combining multiple features. For ST, the use of more than one feature increases the prediction performance (significant one-sampled *t*-test for number of two, three, four, five features); however, there is no further benefit in using more than two features for the prediction (Friedmann test: x^2^ (3) = 5.19, *p* = 0.159). *p* Values were false discovery rate corrected. *p < 0.05; **p < 0.01; ***p < 0.001. Detailed statistics in the Supplementary Material. [Color figure can be viewed at www.neuromodulationjournal.org]

**Figure 6:**
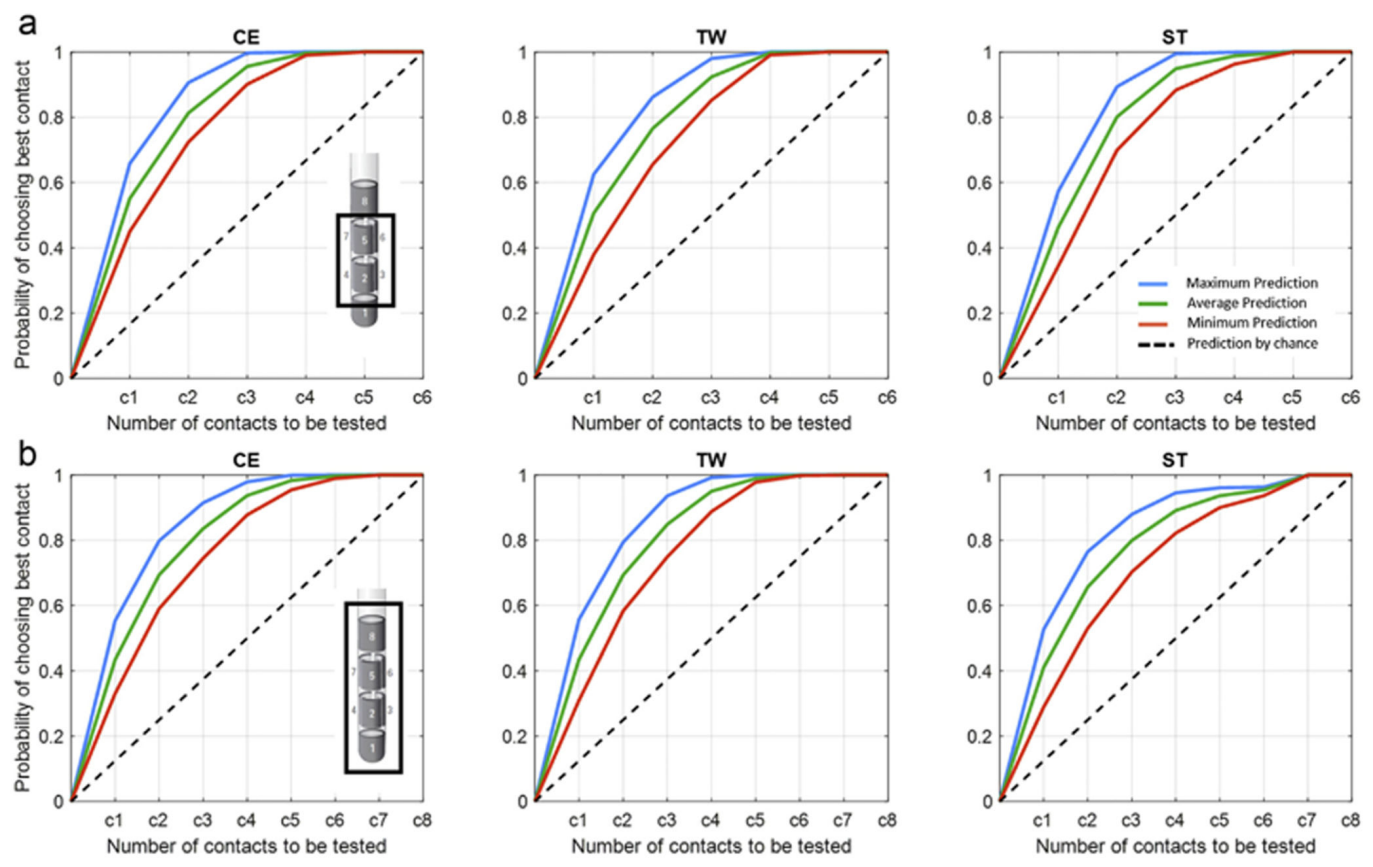
LFP-based prediction of the clinically best upper 30 percentile contacts. Illustrates the output of the second step of the contact prediction method, corresponding to the probability of identifying one of the best stimulation contacts for the three clinical parameters (CE,TW, ST) of the six segmented contacts (a) and of all eight contacts (b). In contrast to Figures 4a and 5a, in which the probability of identifying the highest ranked stimulation contact is illustrated, the current figure illustrates the probability of identifying one of the upper 30 percentile of the best contacts. Within each subplot, the maximum, average, and minimum prediction accuracies evaluated on the hold-out set of nine hemispheres are illustrated as mean value, averaged across the multiple iteration of the prediction pipeline (Fig. 1). The dashed black lines illustrate the prediction by chance (conventional gold standard test strategy), where the probability of identifying the most efficient stimulation contact increases by 0.17 (in a) and 0.125 (in b) after each contact tested. By applying the LFP-based contact prediction strategy, after considering up to half of the electrophysiologically ranked stimulation contacts, the probability of identifying one the best stimulation contacts can reach a maximum as follows: for segmented contacts (a) EF: 99.7%, TW: 98.0, and SE: 99.5%; for all contacts (b) EF: 97.9%, TW: 99.2%, and SE: 94.6%. [Color figure can be viewed at www.neuromodulationjournal.org]

The overall prediction performance following our prediction pipeline (Fig. 1c) is illustrated as maximum, average, and minimum likelihoods in identifying one of the best rated clinical contacts evaluated on the hold-out set of nine hemispheres. It should be noted that the ranking of the contacts is determined by the algorithmic selection and combination of up to five of the best weighted electrophysiological features. When the segmented contacts are considered (Fig. 4a), the probability of identifying the best stimulation site after including the first contact can reach up to 46% for CE, 48% for TW, and 45% for ST. If half of the contacts are included (three out of six), the predictive value can increase to 88% for CE, 85% for TW, and 86% for ST. For the entire DBS lead (Fig. 5a), the maximum probability of identifying the best stimulation site after considering the first contact is around 36 % for CE, 40% for TW, and 36% for ST. By including half of the contacts (four of the eight), the prediction increases to 83% for CE, 86% for TW, and 87% for ST. The prediction performance is clearly above the prediction by chance (current clinical practice). Moreover, by applying less stringent criteria (ie, upper 30 percentile of the clinical ranking) (Fig. 6), the prediction can further be improved, hereby approximating 100% after considering half of the contacts. In addition, Supplementary Data Figure S3 illustrates the clinical performance metric for the CE, TW, and ST of the highest ranked contact using the presented algorithmic approach, which is superior to a contact selected by chance.

### Impact of Combining Multiple Features

The question arises whether the obtained prediction performance in Figures 4a and 5a is only a consequence of the regression-based selection of the single best feature or whether the hierarchical (high to low ranked) combination of multiple features (up to five features) further drives the prediction performance. To answer this, we calculated the percentage change in prediction performance relative to the use of the single highest ranked feature, after stepwise including up to a total of five features. Here, we illustrate two aspects: first, whether there is an incremental or decremental trend in prediction performance across stepwise adding of features (Friedmann test), and second, whether the different feature combinations are different from using a single best feature (one-sampled *t*-test). Figures 4b and 5b refer to the probability of prediction of half of the contacts to be tested (ie, 3/ 6 for segmented contacts and 4/8 for all contacts). In addition, Supplementary Data Figure S4 provides the % change in prediction using the whole contact range. Regarding segmented contacts (Fig. 4b) for CE, adding additional features leads to decrement in contact prediction (x^2^ (3) = 36.26, *p* ≤ 0.001), with all feature combinations performing less than the single best feature. However, despite the finding that using additional features is not further improving the prediction of CE in respect to the use of the single best feature, all feature combinations still allow the prediction of contacts with a clinical metric that outperforms the prediction by chance (Supplementary Data Fig. S3). In contrast, for both TW and ST, there is a significant incremental trend in the prediction performance (TW: x^2^(3) = 36.88,*p* ≤ 0.001;ST: x^2^ (3) = 28.94, *p* ≤ 0.001), with different feature combinations (TW: two, three, four, and five features; ST: three, four, five features) performing better up to 14.2% (TW, combination of five features) and 7.8% (ST, combination of five features) than the single best feature. Similarly, when all contacts are considered (Fig. 5b), there is again a negative trend for CE (x^2^ (3) = 53.94, *p* ≤ 0.001), which is significant for two, three, four, and five features. The prediction of TW again benefits from adding multiple features (x^2^(3) = 50.25, *p* ≤ 0.001), and the combination of two, three, four, and five features shows a significant difference relative to the use of a single feature. For ST, there is no significant incremental trend for using multiple features (x^2^ (3) = 5.19, *p* = 0.159); however, all feature combinations, two up to five, show a significant higher prediction performance than using a single feature. For both TWand ST, the prediction reaches maximum with the combination of four features (TW 14.3% increase, ST 7.4% increase).

### Algorithmic Approach in Comparison to Beta Activity as Single Feature

**Figure 7:**
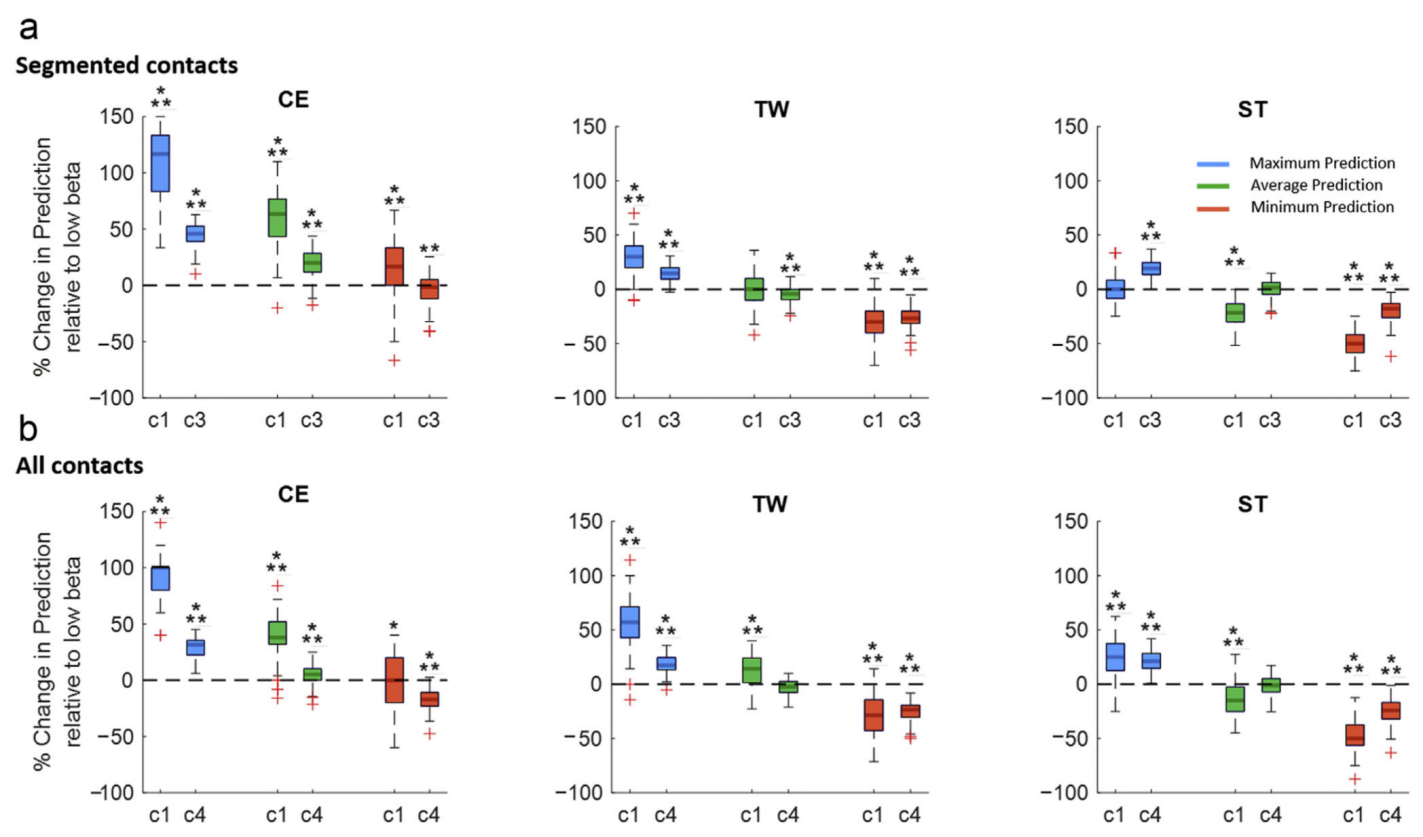
Algorithmic contact prediction vs beta activity as single feature. This illustrates the percentage change of the prediction performance of the presented algorithmic approach relative to the use of beta activity as single feature. The relative change of the prediction performance has been derived as change in the area under the curve for testing only one and up to three contacts out of six segmented contacts (a) as well as for testing one and up to four out of all eight contacts (b). The maximum algorithmic prediction significantly outperforms the use of beta activity for the first-choice contact (1/6: CE [117%] and TW [30%]; 1/8: CE [99%], TW [57%], and ST [25%]) and for half of the contacts (3/6: CE [46%], TW [15%], and ST [20%]; 4/8: CE [32%], TW [17%], and ST [21%]). The average algorithmic prediction outperforms the use of beta activity for first-choice contact (1/6: CE [63%]; 1/8: CE [38%] and TW [14%]), and for half of the contacts (3/6: CE [20%]; 4/8: CE [5%]). The minimum algorithmic prediction outperforms beta activity for the first-choice contact (1/6 CE [17%]). In the remaining iterations, the algorithmic approach does not show an advantage over beta activity used as a single feature. Improvement is illustrated as % median improvement. Statistical comparison was performed as a one-sampled *t*-test (false discovery rate corrected) for all iterations to test whether the prediction is significantly above or below zero (zero corresponds to the prediction obtained by low beta activity). **p < 0.01; ***p < 0.001. Detailed statistics in the Supplementary Material. [Color figure can be viewed at www.neuromodulationjournal.org]

As additional subanalyses, we evaluate to what extent the presented algorithmic approach can outperform low beta activity used as a single feature, which currently represents the single best validated symptom biomarker for PD (Fig. 7). The relative change of the prediction performance has been derived as change in the area under the curve for testing only one and up to three contacts out of six segmented contacts (Fig. 7a) as well as for testing one and up to four of all eight contacts (Fig. 7b). The maximum algorithmic prediction outperforms the use of beta activity as a single feature with a significant median percentage improvement for the first-choice contact (1/6: CE [117%] and TW [30%]; 1/8: CE [99%], TW [57%], and ST [25%]) and for half of the contacts (3/6: CE [46%] TW [15%], and ST [20%]; 4/8: CE [32%], TW [17%], and ST [21%]). The average algorithmic prediction outperforms the use of beta activity with a significant median percentage improvement for first the choice contact (1/6: CE [63%]; 1/8: CE [38%], and TW [14%]) and for half of the contacts (3/6: CE [20%]; 4/8: CE [5%]). Finally, the minimum prediction obtained by the algorithmic approach significantly outperforms beta activity as a single feature in the following situation, the first-choice contact (1/6 CE [17%]). In the remaining iterations, the algorithmic is equally- or underperforming beta activity used as a single feature. Moreover, Supplementary Data Figure S5 illustrates the entire contact prediction plot based on low beta activity.

### Inference of Chronic Stimulation Setting

To study whether the contact prediction obtained with the current approach is still informative for the chronic stimulation setting, we repeated the analysis using the documented stimulation setting at 12.5 months (instead of 5.4 months) postoperatively. In this cohort, most patients have a ring mode-stimulation in chronic use, and the stimulation level is usually selected according to the most convenient TW. Thus, we investigated if the "best LFP contact," defined by the spectral features for TW, can indicate the chronic stimulation level in use. This shows that the best chronic level can be identified in 47.3% after including one contact and further increases to 78.1% after including two contacts (Supplementary Data Fig. S6), which are both beyond the prediction by chance.

### Combining Electrophysiological Features With an Anatomical Stimulation Hotspot

**Figure 8:**
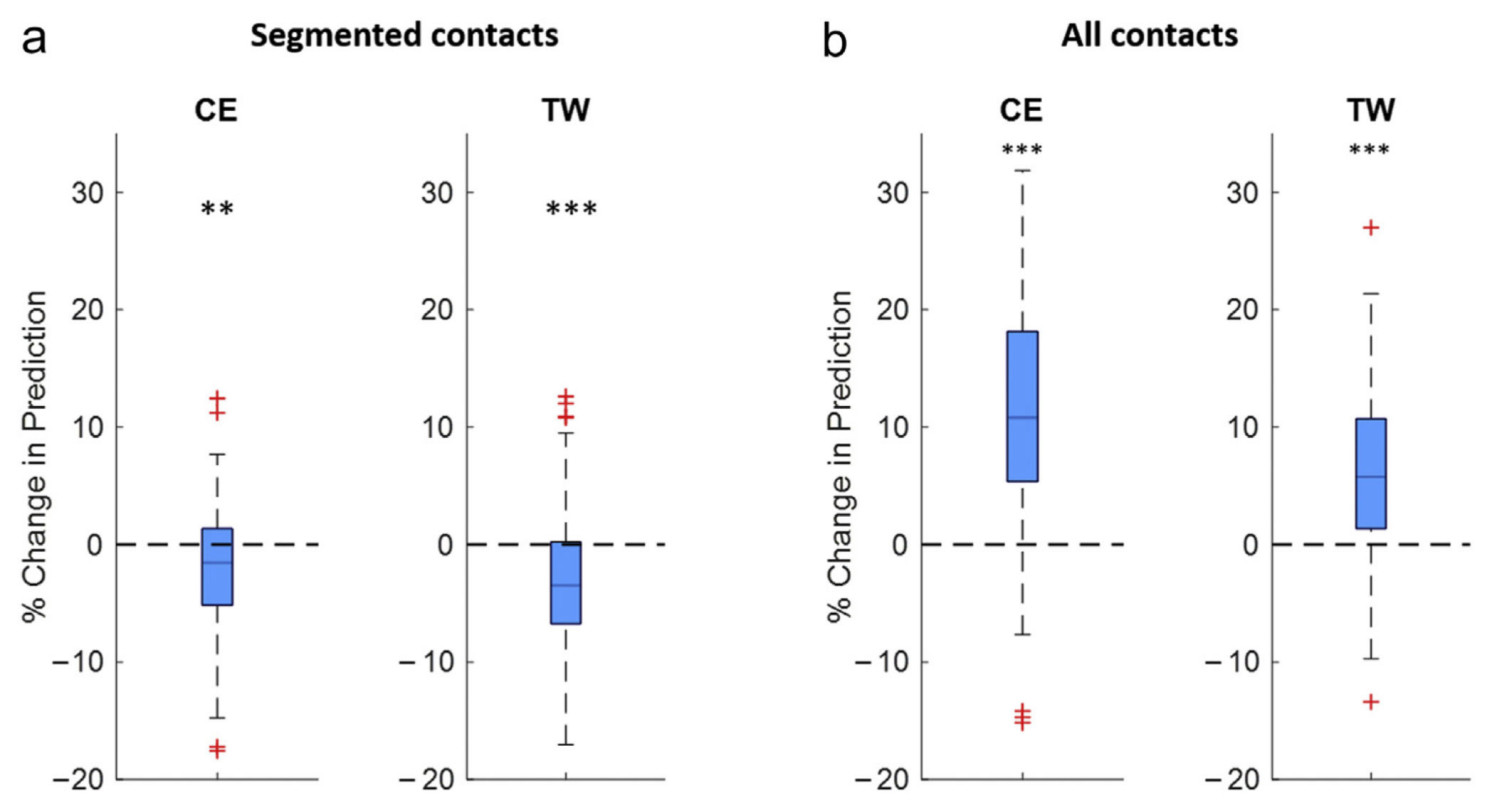
LFP-based contact prediction combined with anatomy. This illustrates the percentage change of the prediction performance when adding an anatomical landmark of the dorsal STN (ie, stimulation sweet spot for low stimulation ET) as feature to the prediction algorithm. The prediction performance is again illustrated as change in the area under the curve for the first three of six segmented contacts (a) and four of eight contacts (b) of the combined approach (LFP + anatomical landmark) relative to the LFP approach alone. This anatomical reference point has been validated on the ET, used to calculate both CE and TW; therefore, results are indicated for those two clinical parameters. When only the six centered segmented contacts are considered (a), adding the anatomical landmark as feature to the prediction leads to a slight deterioration of the prediction performance without beneficial effect in most instances (median: CE: −1.5, TW: −3.48%). When the entire DBS lead is considered (b), combining LFP features with the anatomical landmark leads to a significant increase of the prediction performance (median: CE: 10.8%, TW 5.8%). One-sampled *t*-tests (false discovery rate corrected); **p < 0.01; ***p < 0.001. Detailed statistics in the Supplementary Material. [Color figure can be viewed at www.neuromodulationjournal.org]

To investigate whether LFP-based contact prediction can be refined through anatomical information, we included an anatomical reference point, sweet spot distance (SSD), into the feature pool of the algorithm (Fig. 1c). SSD is defined as the minimum distance of the contact's center to a previously published VTA-based anatomical reference point that indicates a low stimulation ET within the motor STN.^30^ This reference point has been validated on the ET, used to calculate both CE and TW; therefore, results are indicated for those two clinical parameters. The results show that adding this anatomical landmark as feature does not improve the prediction performance for the six segmented contacts (Fig. 8a; median: CE: −1.5, TW: −f3.48%). In contrast, when the entire DBS lead is considered (Fig. 8b), adding the anatomical landmark leads to an increase of the prediction (median: CE: 10,8%, TW 5.8%). Supplementary Data Figure S7 illustrates the entire contact prediction plot solely based on the anatomical hotspot.

## Discussion

This study demonstrates the potential of algorithmic selection and combination of multimodal features to guide multicontact DBS programming. We derive four major findings from this work. First, LFPs directly recorded from the DBS lead can be used to predict the optimal stimulation contacts for different clinical DBS outcome parameters (CE, TW, ST), all with a similar prediction performance. Second, algorithmic selection and combination of resting and movement state spectral features have the potential to outperform the use of beta activity as single feature. Third, the LFP-based method can predict the chronically used stimulation setting. Fourth, combining electrophysiological features with anatomical markers can further refine the prediction of the optimal stimulation site.

### LFP-based Programming

The clinical use of multicontact DBS leads is more complicated and time-consuming because of the myriad of contact-combination^6^ and the lack of supportive tools, which hinders the efficient exploitation of this technology.^34^ In this study, we demonstrate that LFPs recorded from the DBS target structure could effectively be used to short-list and select optimal stimulation contacts, which would allow the clinician to focus on fewer but more promising stimulation contacts to be used alone or in combination. Indeed, by considering just half of the best ranked stimulation contacts, the prediction accuracy can reach almost 90%, thereby saving half of the programming time. At the same time, this result illustrates that none of the features is 100% predictive; otherwise, maximum accuracy would be reached after considering the highest electrophysiological ranked contact. For the interpretation of our presented method, it is important to note that we aim to predict the best rated clinical contact, which, although being a strict definition, serves to approximate the performance of LFP-based contact prediction. However, for completeness, we have also investigated the value of our method when applying a more relaxed definition (ie, prediction of a contact that is among the top 30% of best contacts). This is because, in clinical practice, more than one contact can be sufficient (alone or in combination) to help control symptoms, and therefore, an algorithmic approach that can identify any contact among the relatively better contacts is still anticipated to be clinically relevant and useful. Promising is the finding that LFP-based programming could be a versatile practical tool because a high prediction performance is reached independently of whether the desired clinical outcome parameter is CE, TW, or SE or whether segmented or all (ring + segmented) contacts are considered. Thus, the data presented here can be informative for different lead implantation and programming routines applied in the different DBS centers. Moreover, the LFP-guided contact selection can predict the level used for chronic DBS at 12.5 months after surgery

### Selection and Combination of Features

An important novelty of this work is the algorithmic selection and combination of multiple resting and movement state spectral features to inform about the optimal stimulation contacts. Of interest is the observation that contacts exhibiting less gamma power are more predictive for high clinical efficacy and broad TW. Gamma power is broadly seen as prokinetic, whereas resting beta activity is rather indicative for motor impairment such as bradykinesia and rigidity, and the current results extend this inverse functional relationship to the framework of DBS contact prediction. In addition to resting-state features, movement-state features such as beta ERD and HFO ERS also turned out to be informative for the ST. For beta ERD, we argue that movement-related modulation should generally be indicative for the motor area of the target structure,^19^ whereas a larger distance to this modulation hot spot could indicate the proximity to neighboring structures, which, if stimulated, can induce side effects. The path-ophysiological role of HFOs is less well understood, but like previous studies, we found a negative clinical-electrophysiological relationship.^24,25^

Previously, electrophysiology-based strategies to improve DBS programming mainly involved STN beta activity,^12–15,20,35^ and studies already showed very promising results with a varying degree in contact prediction performance. Although beta activity is one of the most predictive features, with the current algorithmic approach, the prediction performance can be substantially increased. It also revealed that for CE, the data-driven selection of a single feature already reaches the maximum prediction of the best stimulation contact in most cases and adding further features does not add value, whereas the prediction of TW or ST clearly benefits from the combination of multiple features. A partial explanation for this could be that both TW and ST are slightly more complex parameters depending on more clinical-anatomical properties than CE. To increase the robustness of contact prediction algorithms, as well as to refine the number and weights of predictive features, studies on larger data sets are necessary.

### Alternative and Complementary Methods

Beyond the spectral features used in this work, there are other promising electrophysiological markers for DBS contact prediction on the horizon. Recently, attention has been drawn to evoked resonant neural activity (ERNA), which can localize in the dorsal subregion of the STN and even be measured during general anesthesia.^36–38^ Measuring ERNA requires a high recording sample rate and a particular stimulation/recording paradigm, and is currently limited to an intraoperative recording set-up. In the future, the electrophysiological assessment for contact site prediction should be performed in the postoperative state using novel neurostimulators with brain sensing capabilities,^39^ and such devices should optimally be adjusted to enable recording for the full spectral content, including HFO and evoked activities. This would allow for a more flexible functional-electrophysiological profiling in the chronically implanted patient without the limitation of the intraoperative stun-effect and time constraints.

Another commonly investigated and intuitive approach to detect the optimal stimulation site is based on imaging, particularly on VTA modeling.^40^ This can be used to establish probabilistic outcome maps by aggregating the VTAs in large patient populations, as well as being linked to the network level by integrating fiber tract activations.^31,41–43^In the current work, we demonstrate that already adding an anatomical landmark indexing a sweet spot in the dorsal motor region to the feature list can refine the prediction. This is clear when the optimal stimulation contact is predicted from the entire span of the DBS lead, but less so when only the six centered segmented contacts are considered. The difference in the anatomical-spatial extent and spatial resolution of the two contact groups might explain this finding and may also partially contribute to the differences in the ranking of features and prediction scores, whereas other factors giving rise to spectral differences still need to be investigated. Beyond contact selection, DBS programming involves tuning of other parameters such as stimulation amplitude, frequency, and pulse width. To predict the latter three parameters for individual patients based on LFPs is challenging because this would require investigating the electrophysiological response curves during systematic stimulation with a broad range of stimulation parameters, an endeavor that must be investigated in future work. Imaging-based methods could provide another promising avenue because stimulation parameters could be approximated by stimulation field modeling combined with functional anatomy. Although the exact biophysical properties of both electrophysiological and imaging methods still need to be investigated and better understood, the current work crystallizes the idea that the combination of spectral and imaging features can be a powerful tool to explain more of the variance in clinical DBS response and to better guide DBS programming in the future.

### Limitation

The LFP-based contact prediction performance shown here could be underestimated because the algorithm used determines the likelihood of identifying the single best stimulation contact, whereas in clinical practice, the combination of contacts is often used to reach optimal symptom control. Also, the clinical assessment was limited to upper-limb rigidity testing, which is, however, the most sensitive and reliable clinical sign for systematic DBS programming and a good proxy for bradykinesia symptoms.^2^However, the optimal stimulation contact and parameters for controlling other motor symptoms such as tremor and gait or nonmotor symptoms can be different and would therefore need to be adjusted on a case-by-case basis. Future studies need to investigate electrophysiological and anatomical programming markers to guide the optimization according to individual symptom profiles.

Moreover, manual clinical contact testing is a subjective method, and noise in the assessment may only have led to degrading the apparent predictive value of LFPs. We also assumed that lead position and orientation did not change after placement, which is also supported by recent literature.^44^ Any such rotation, however, might again have rather diminished the prediction performance. To prevent the impact of voltage differences in the raw signal owing to different biophysical properties of the contacts or variability in impedances, our processing included the normalization (z-scoring) of the filtered raw signal. Overall, applying normalization improves the comparability of bio-signals; however, we cannot exclude that in single instances, normalization might have led to some spectral distortion and suboptimal representation of power values, which we have tried to minimize by high-pass filtering the data at 5 Hz before any normalization. We also acknowledge that in computational pipelines as used in this work, overfitting is a challenge, and several methodologic steps (such as regularization via Lasso, hold-out set for testing, etc) have therefore been implemented to minimize it. In addition, to ensure a unified input distribution, clinical and electrophysiological features were normalized within hemisphere and feature category before feeding into Lasso regression. There is now a need to externally validate the proposed method by using a separate independent population cohort, and a prospective study design would also be needed. Moreover, the LFP signal-to-noise ratio may have been reduced because of stun effects, which can be detected as the STN is traversed.^45^

## Outlook and Conclusion

In conclusion, this study suggests that a data-driven selection and combination of movement- and resting-state features from LFPs can be informative to optimize DBS programming in PD. Future work of this kind should include larger patient cohorts to increase the robustness of features selection and to set the foundation for user-friendly programming algorithms. The recent introduction of neurostimulators with brain sensing capabilities will facilitate systematic and widespread LFP measurements across different DBS indications. Similar approaches can also be used to determine the optimal LFP recording sites for closed-loop DBS algorithms. Overall, there is little doubt that these and other digital solutions, likely in combination, will optimize the use of DBS in clinical practice in the future.

## Supplementary Material

To access the supplementary material accompanying this article, visit the online version of *Neuromodulation: Technology at the Neural Interface* at www.neuromodulationjournal.org and at https://doi.org/10.1016/j.neurom.2022.01.017.

Supplementary material
