# Supplementary material for: Combining Multimodal Biomarkers to Guide Deep Brain Stimulation Programming in Parkinson Disease
Source: Neuromodulation. Author manuscript; Available in PMC 2023 Feb 4. (PMC7614142; doi:10.1016/j.neurom.2022.01.017)
Supplement: Supplementary material [file EMS144707-supplement-Supplementary_material.docx]

**Supplementary material**

**Combining multimodal biomarkers to guide deep brain stimulation programming in Parkinson’s disease**

**Methods:**


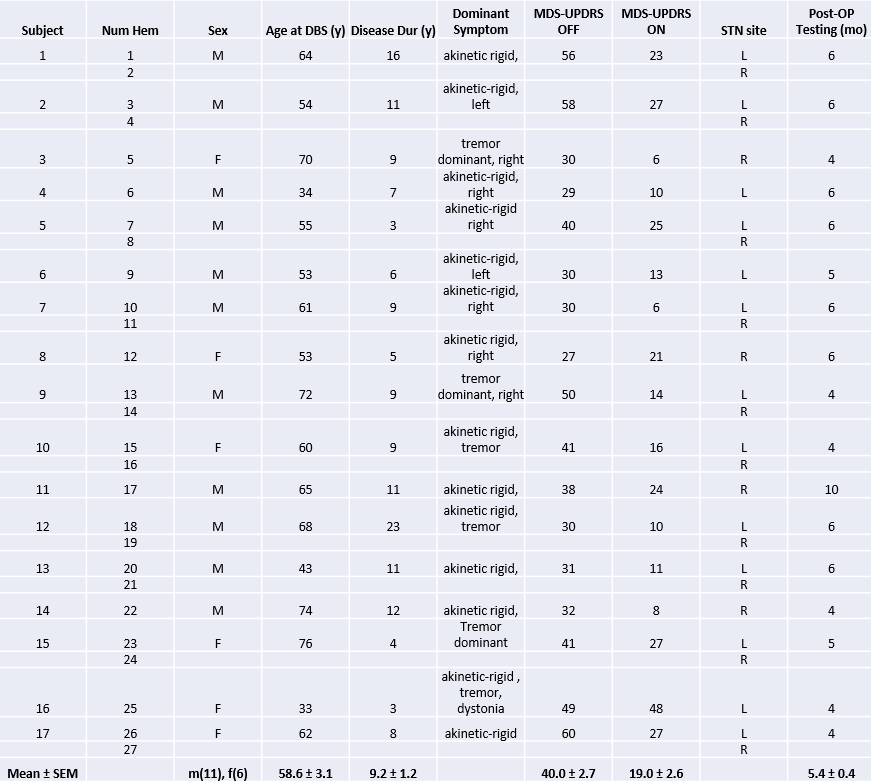


**Supplementary Table 1: Clinical details.** Num = number; Hem = hemisphere; R = right; L = left; y = years; mo = months; M = male; F= female; MDS-UPDRS = Movement Disorder Society - Unified Parkinson’s disease rating scale Part III; SEM = standard error of the mean;

**DBS surgery**

Routine dopaminergic medication was stopped before the surgery. Preoperative 3T MRI and stereotactic CT with Leksell G frame were used in Brainlab iPlan 3.0 Stereotaxy software to localize the STN. During the awake surgery, the target selection was optimized using microelectrode recordings and selective test stimulation. The Boston Vercise Cartesia directional leads (Boston Scientific) were implanted in both hemispheres of the patients. These electrodes have a hybrid contact geometry: the top (ring) and the bottom (bullet tip) level are contacts for omnidirectional stimulation and the two levels in the middle have three segmented contacts to enable directional/focused recording and stimulation in three different directions (angled at 120° each) (Figure 1A).

## Anatomical contact localization

Lead reconstruction was performed with the Lead-DBS toolbox (version 2.3.2) in Matlab 2019b (The Mathworks, Natick, MA, USA) as we and other groups have described recently[1, 2]. Preoperative magnetic resonance images and postoperative computer tomography images were co-registered and normalized into the Montreal Neurological Institute (MNI) space (MNI152 NLIN 2009b) using Statistical Parametric Mapping 12 and Advanced Normalization Tools [3]. A brainshift correction was applied with the coarse and fine mask [1, 4]. Each co-registration and normalization was checked manually before further processing. The electrode trajectory and position were reconstructed semi-automatically using PaCER and corrected manually, when necessary [5]. Lead orientations were estimated with the DiODe algorithm and double checked with post-operative X-rays [6]. The location of each contact is characterised by its xyz-coordinates in the MNI space, which were projected on the anatomical STN from the Distal atlas [7].

**Features selection, contact prediction and statistical analyses (detailed description).**

All statistical analyses were performed using MATLAB (2019b, Mathworks, Natick, MA, USA). To study the predictive value of the spectral features, a two-step strategy was used: feature selection followed by prediction logic (see method figure 1C):

*Feature selection:*

Regularization is a widely used technique that ensures that any model developed is generalizable by reducing the chances of overfitting. In this work, we have specifically chosen lasso for regularization because it can simultaneously aid in feature selection by forcing unimportant features to have a coefficient of zero (effectively “knocking” them out of the trained model) [9]. This latter step additionally handles the multi-collinearity which may exists between dependent features. The input for the lasso consisted of clinical and electrophysiological features, which were first z-scored within feature category and hemisphere, to not bias the feature selection towards lower frequencies and to ensure a unified distribution. Secondly the normalized clinical and electrophysiological data were concatenated to one large data matrix. Within lasso, the amount of regularization is controlled by a parameter, λ. By using an iterative approach, we can test large number of values with varying degrees of regularization and then choose the model that first revealed the best 10 features. In this work, we have used 5-fold cross validation when selecting the optimal model. A 5-fold cross validation effectively provides us with 5 different “optimal” models, one for each fold.

*Prediction logic:*

The prediction-logic applied here was developed in-house and has been published before [10]. In summary, the goal of this algorithm is to determine the probability of identifying the best stimulation contact for a given clinical parameter (CE, TW or ST) by increasing the number of contacts to be tested. The order of the contacts to be tested is either by chance (clinical practice) or informed by the predictive value of the LFP features and we therefore distinguish these 2 scenarios: 1. *Prediction by chance*: This refers to the clinical gold standard, the trial- and error-based method. Assuming a DBS lead with 8 contacts, after each contact tested, the probability of identifying the best stimulation contact increases for 0.125 and reaches 1 (=100%) once all 8 contacts have been tested. 2. *LFP-based prediction*: Probability of identifying the best stimulation contact depending on the LFP-based ranking of the contact. The LFP-based ranking was established by ranking the contacts according to the best weighted feature out of the lasso, i.e. *feature1 x weight1* and the resulting ranking was used to predict the clinically best stimulation contact. The same process was iterated multiple times by including the next best features $feature 1\times weight 1+feature 2\times weight 2 + ... + feature n\times weight n$ , updating the ranking and repeating the contact prediction.

The above two-step strategy was applied to different subsets of the dataset i.e. out of the 27 hemispheres, 18 hemispheres (i.e. 66.7 % of data) were used to determine the feature weighting and ranking using lasso regression with 5 fold cross-validation and 9 hemispheres (i.e. 33.3% of data) were used for the prediction logic (as described above). Importantly, the data set for prediction corresponds to a hold-out data set and was not used during the feature selection stage. This step was performed 3 times, having each block of 9 hemispheres once part of the testing data with the remaing block of 18 hemispheres for training. To further mitigate the risk of certain hemispheres driving results within each block and thus to increase the generalisability of the results, we implemented 100 more iterations with randomized allocation of the hemispheres to each block. For each of the 100 repetitions we extracted and reported the maximum, average and minimum prediction obtained using different feature combination obtained using different combinations up to a maximum of 5 features.

Further correlation analyses were performed using non-parametric Spearman correlation. Note, the analyses pipeline was executed twice, once considering the six centered segmented contacts only (in the text referred as “segmented contacts”) and once considering all eight contacts (in the text referred as “all contacts”). This distinction was made as segmented and ring contacts might differ in their biophysical properties, spatial resolutions and clinical response to stimulation. Multiple comparisons were corrected using FDR (false discovery rate). All data are presented as means ± standard error of the mean (SEM) or standard deviation (std) as indicated in the legends.

**Results:**

**Resting and movement state spectral curves**

**
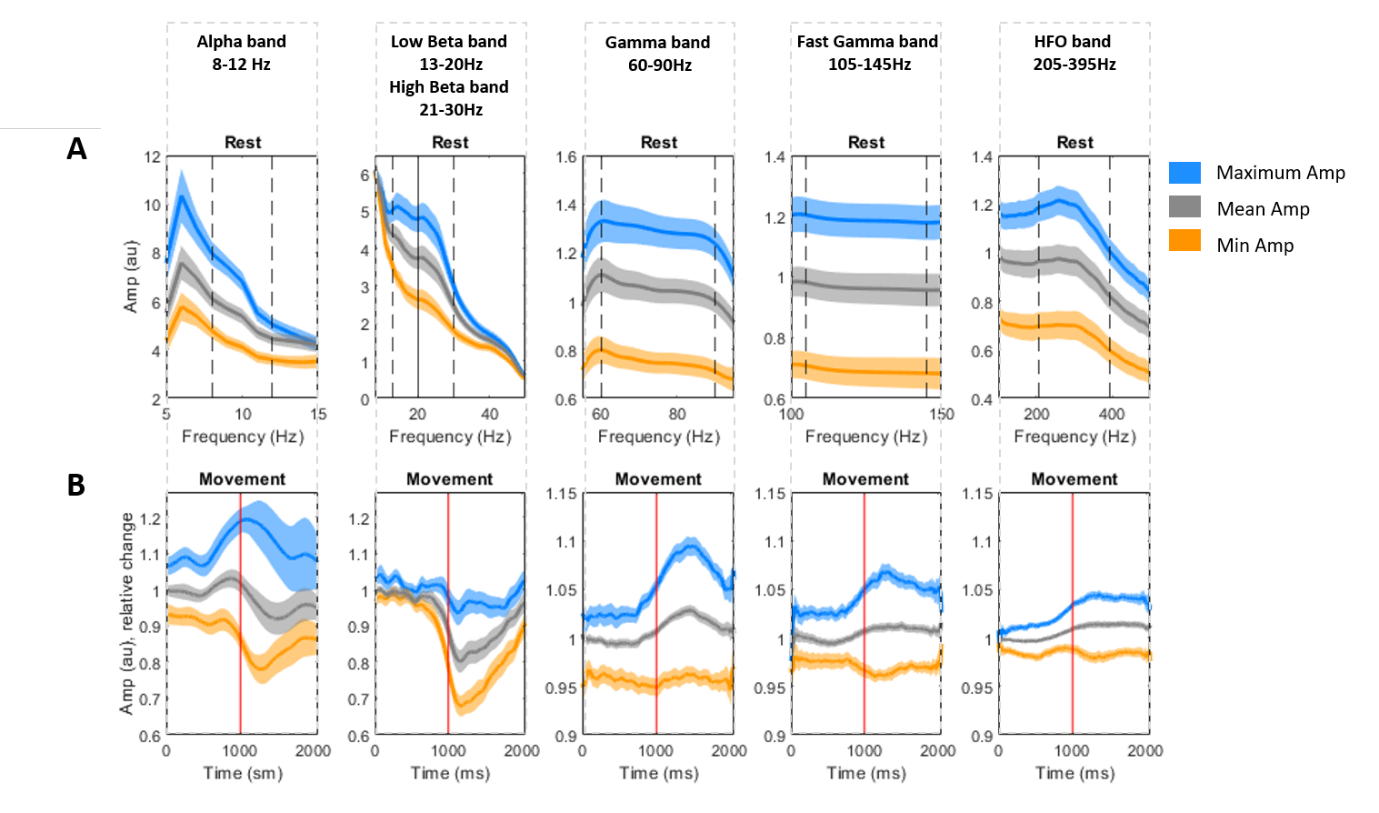
**

**Supplementary figure 1:** Illustrates the resting state amplitude frequency plots (A) and the movement state event-related spectral modulation plots (B) for the spectral features used in this work for contact-prediction (alpha 8-12Hz, low beta 13-20 Hz, high beta 21-30Hz, gamma 60-90Hz, fast gamma 105-145 Hz and HFO 205-395 Hz). For every single DBS lead we determined three different amplitude values: 1. Contact with maximum amplitude, 2. Mean amplitude across all contacts and 3. Contact with minimum amplitude separately for the resting and movement state. These values have hen been averaged across the 27 hemispheres. Note, for beta activity, both the low and high beta frequency band have been illustrated in one plot. The red vertical line in B illustrates the onset of the movement. (see method section on signal processing for additional, relevant details).

**Spectral feature relationship**

A prerequisite of combining multiple features for the contact prediction is a putative additive rather than redundant information. Supplementary figure 1 illustrates the autocorrelation between the spectral features and within each DBS lead (all eight contacts considered). This reveals a median absolute r-value of 0.11 and median p-value of 0.48, which indicates a low degree of information redundancy across the features used in this work.


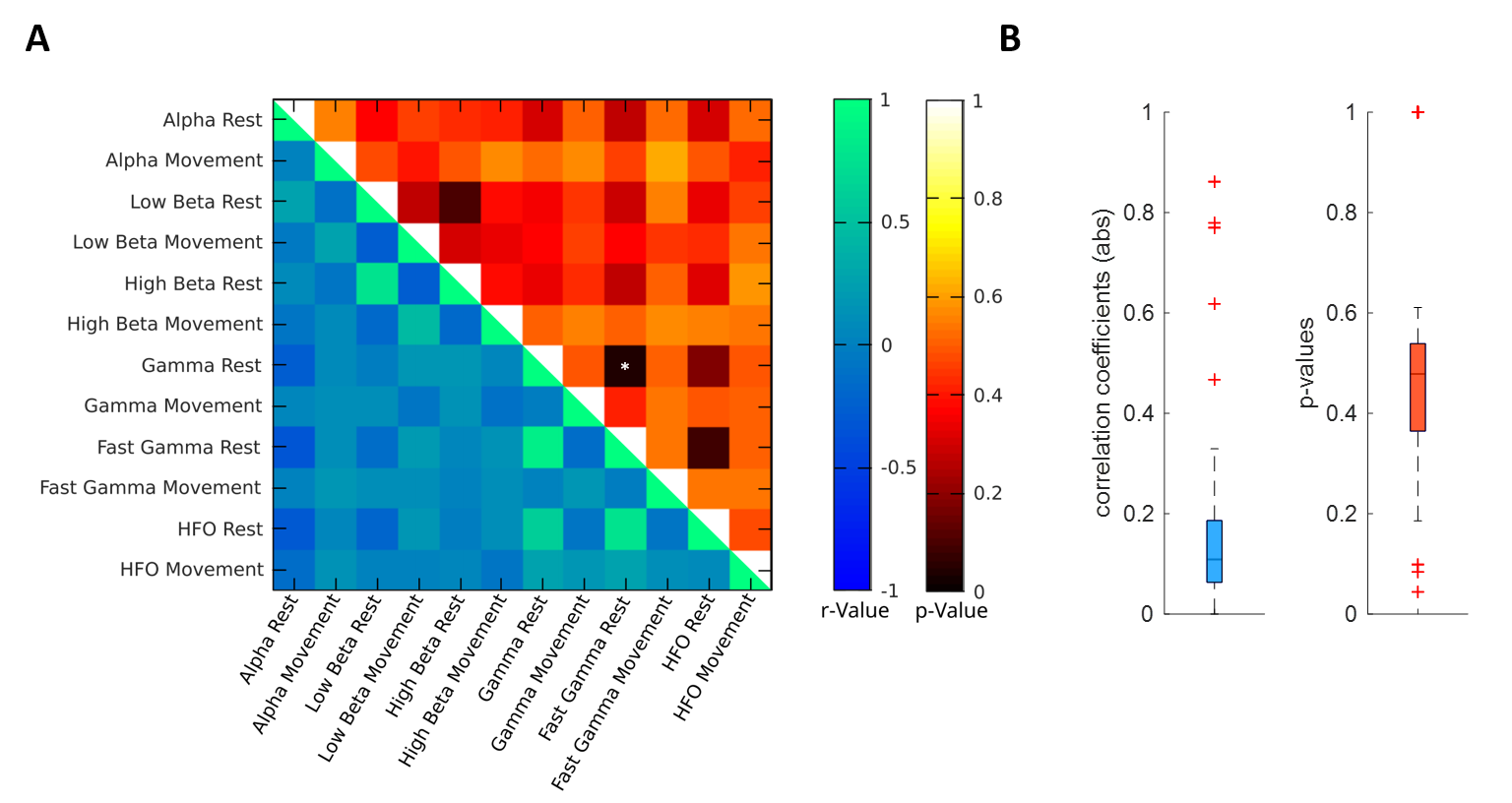


Supplementary Figure 2. **Feature relationship and within DBS lead**. **A**: Illustrates the averaged correlation coefficients (blue color scheme, below) and averaged p-values (red color scheme, above) from the Spearman’s correlations between the 12 spectral features (alpha 8-12Hz, low beta 13-20 Hz, high beta 21-30Hz, gamma 60-90Hz, fast gamma 105-145 Hz and HFO 205-395 Hz). Correlations were performed for each DBS lead (n=27) and r-values were then averaged across DBS leads. A significant p-value is only reached by the correlation of gamma rest with fast gamma rest. **B**: Complementary to figure (A), these box plots illustrate the distribution of the absolute r-values (left) and p-values (right) (autocorrelations between the same feature excluded). The median r-value is 0.11 and median p-values is 0.48, which overall indicates a low degree of information redundancy across the different features.


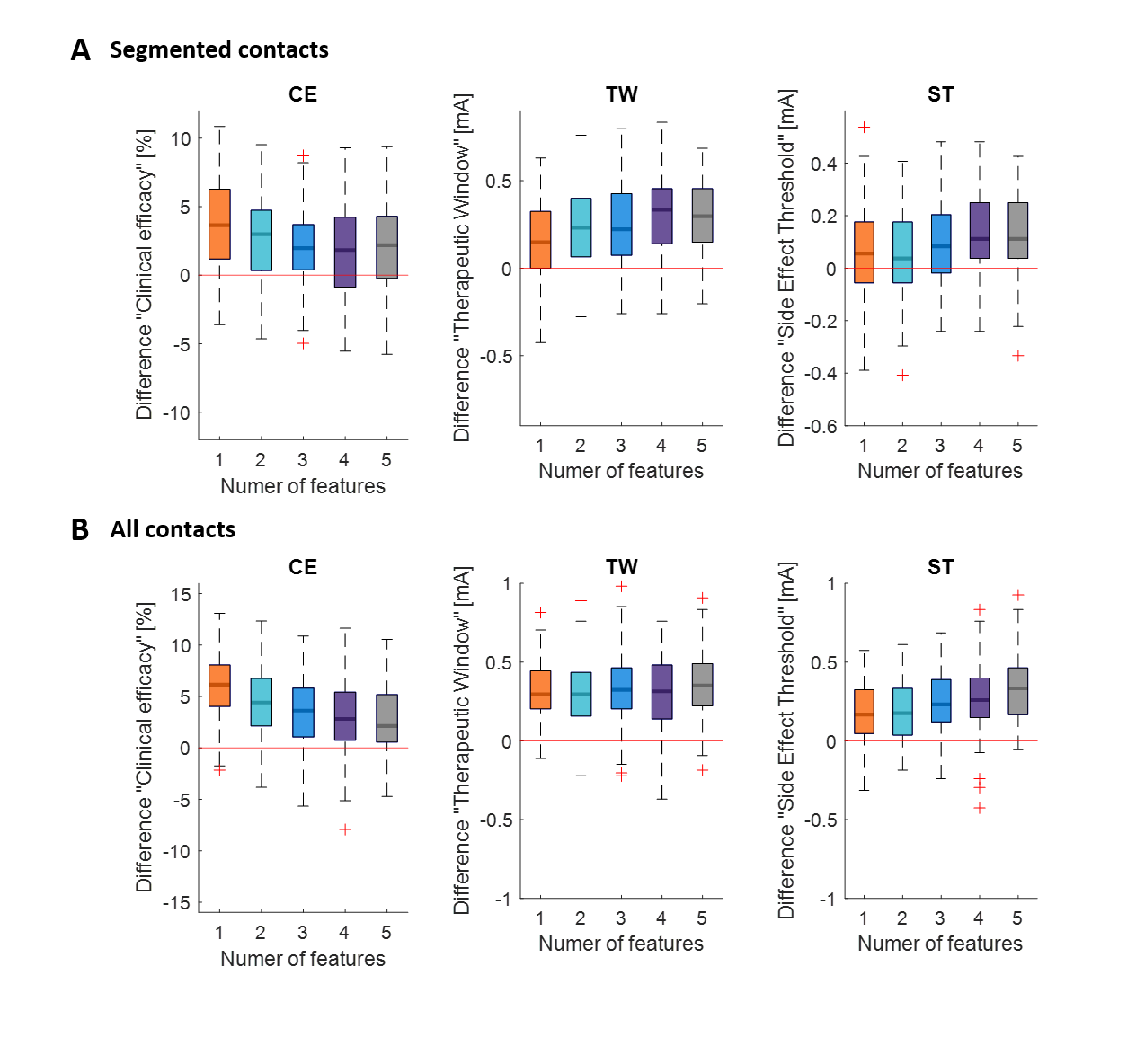


**Supplementary figure 3**: Illustrates the DBS outcome metrics (“clinical efficacy, “therapeutic window”, “side effect threshold”) obtained by the highest ranked electrophysiological contact as difference to the outcome metric obtained by a randomly chosen contact, averaged across hemispheres shown as boxplot for the 100 prediction iterations. The results are shown for segmented contacts (A) and all contacts (B) and using the best ranked electrophysiological feature, up to the combination of 5 features. Note, the clinical metric for therapeutic window and side-effect threshold corresponds to milli Ampere (mA), while the metric for clinical efficacy is in % improvement (see method section). A value of “0” would imply that the clinical outcome obtained with the best ranked contact derived from our prediction pipeline would not be different from a contact chosen by chance (i.e. clinical practice). Values above “0” imply that the contact chosen by the prediction pipeline performs better. Indeed, the results illustrate that the pipeline derived contact provides a significant increase for all three clinical metrics, clinical efficacy (median ranging from 1.8% to 3.6% for segmented and 2.13% to 6.14% for all contacts), therapeutic window (median ranging from 0.14 to 0.33mA for segmented and 0.30mA to 0.35mA for all contacts) and side-effect threshold (median ranging from 0.04mA to 0.1mA for segmented and 0.17mA to 0.34mA for all contacts). Regarding the impact of adding more features, for CE in both segmented (A) and all contacts (B), adding more features leads to a significant decrease in the predicted clinical metric. For TW and segmented contacts (A) adding more features increases the predicted clinical metric, but remains unchanged for all contacts (B). For ST adding more features increases the predicted clinic metric for both contact configurations (A and B). Detailed statistics below in table below. CE: Clinical efficacy, TW: therapeutic window; ST: Side-effect threshold; mA: milli Ampere

**Statistic supplementary figure 3 A**

**Effect size legend**

d=0.01--> very small effect size
d=0.20--> small effect size
d=0.50--> medium effect size
d=0.80--> large effect size
d=1.20--> very large effect size
d = 2.00 --> huge effect size

Clinical efficacy

Friedmann test: x^2^(4) = 19.25, p =0.001

| Comparison | 1 feat | 2 feat | 3 feat | 4 feat | 5 feat |
| --- | --- | --- | --- | --- | --- |
| P-values | <0.001 | <0.001 | <0.001 | <0.001 | <0.001 |
| Effect-size | 1.05 | 0.80 | 0.70 | 0.55 | 0.63 |

Therapeutic Window

Friedmann test: x^2^(4) = 25.36, p <0.001

| Comparison | 1 feat | 2 feat | 3 feat | 4 feat | 5 feat |
| --- | --- | --- | --- | --- | --- |
| P-values | <0.001 | <0.001 | <0.001 | <0.001 | <0.001 |
| Effect-size | 0.73 | 0.99 | 1.02 | 1.34 | 1.40 |

Side effect Threshold

Friedmann test: x^2^(4) = 17.49, p =0.002

| Comparison | 1 feat | 2 feat | 3 feat | 4 feat | 5 feat |
| --- | --- | --- | --- | --- | --- |
| P-values | 0.003 | 0.001 | <0.001 | <0.001 | <0.001 |
| Effect-size | 0.30 | 0.34 | 0.63 | 0.88 | 0.85 |

***Statistic supplementary figure 3 B***

Clinical efficacy

Friedmann test: x^2^(4) = 57.37, p <0.001

| Comparison | 1 feat | 2 feat | 3 feat | 4 feat | 5 feat |
| --- | --- | --- | --- | --- | --- |
| P-values | <0.001 | <0.001 | <0.001 | <0.001 | <0.001 |
| Effect-size | 1.80 | 1.28 | 1.04 | 0.87 | 0.78 |

Therapeutic Window

Friedmann test: x^2^(4) = 5.57, p = 0.234

| Comparison | 1 feat | 2 feat | 3 feat | 4 feat | 5 feat |
| --- | --- | --- | --- | --- | --- |
| P-values | <0.001 | <0.001 | <0.001 | <0.001 | <0.001 |
| Effect-size | 1.80 | 1.31 | 1.57 | 1.23 | 1.60 |

Side effect Threshold

Friedmann test: x^2^(4) = 32.49, p <0.001

| Comparison | 1 feat | 2 feat | 3 feat | 4 feat | 5 feat |
| --- | --- | --- | --- | --- | --- |
| P-values | <0.001 | <0.001 | <0.001 | <0.001 | <0.001 |
| Effect-size | 0.85 | 0.96 | 1.29 | 1.22 | 1.58 |


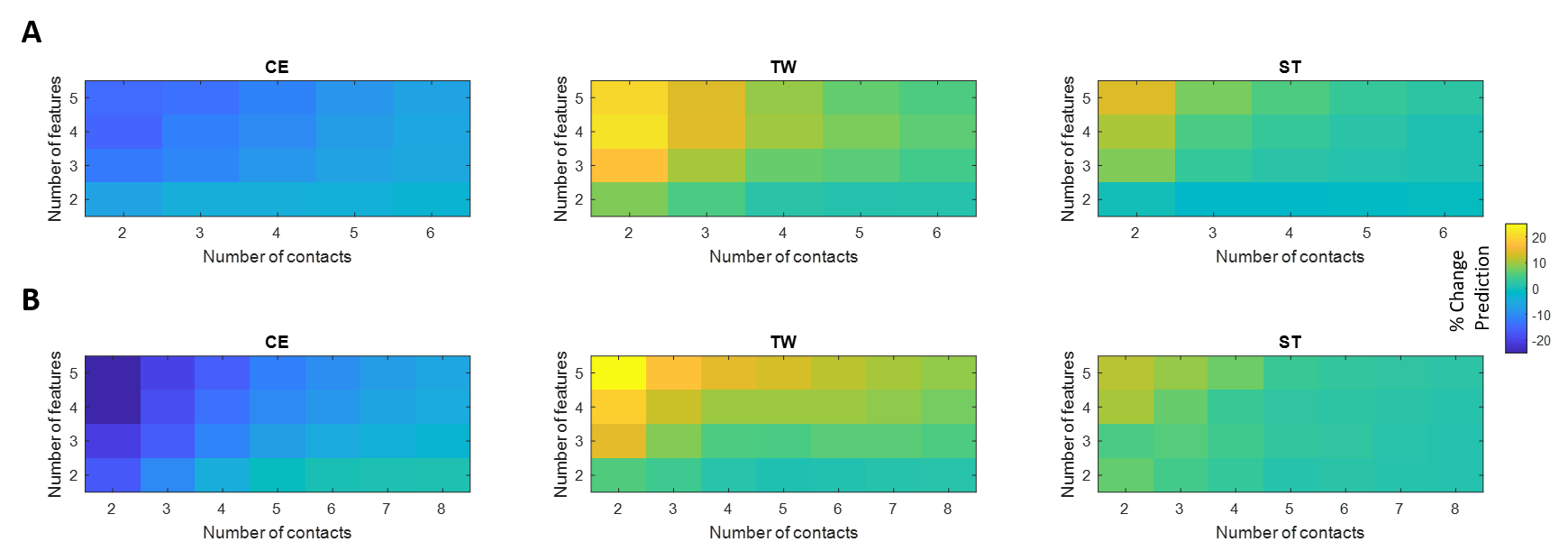


**Supplementary figure 4:** Step-wise change in prediction: Illustrates the percentage change in contact prediction following the combination of multiple features and increasing number of contacts relative to the use of the single highest ranked feature of a single contact to be used. The average change in prediction is illustrated for the three clinical parameters (CE, TW, ST) and for both segmented (A) and all contacts (B). For CE adding additional features overall leads to a reduction of the prediction performance while for TW and ST the prediction performance can be increased by combining multiple features. The effect is stronger for fewer contacts to be used for prediction and saturates toward considering all contacts. Also see figure 4B and 5B in which the example for 3/6 and 4/8 contacts to be used for prediction is illustrated including detailed statistical testing. CE: Clinical efficacy, TW: therapeutic window; ST: Side-effect threshold;


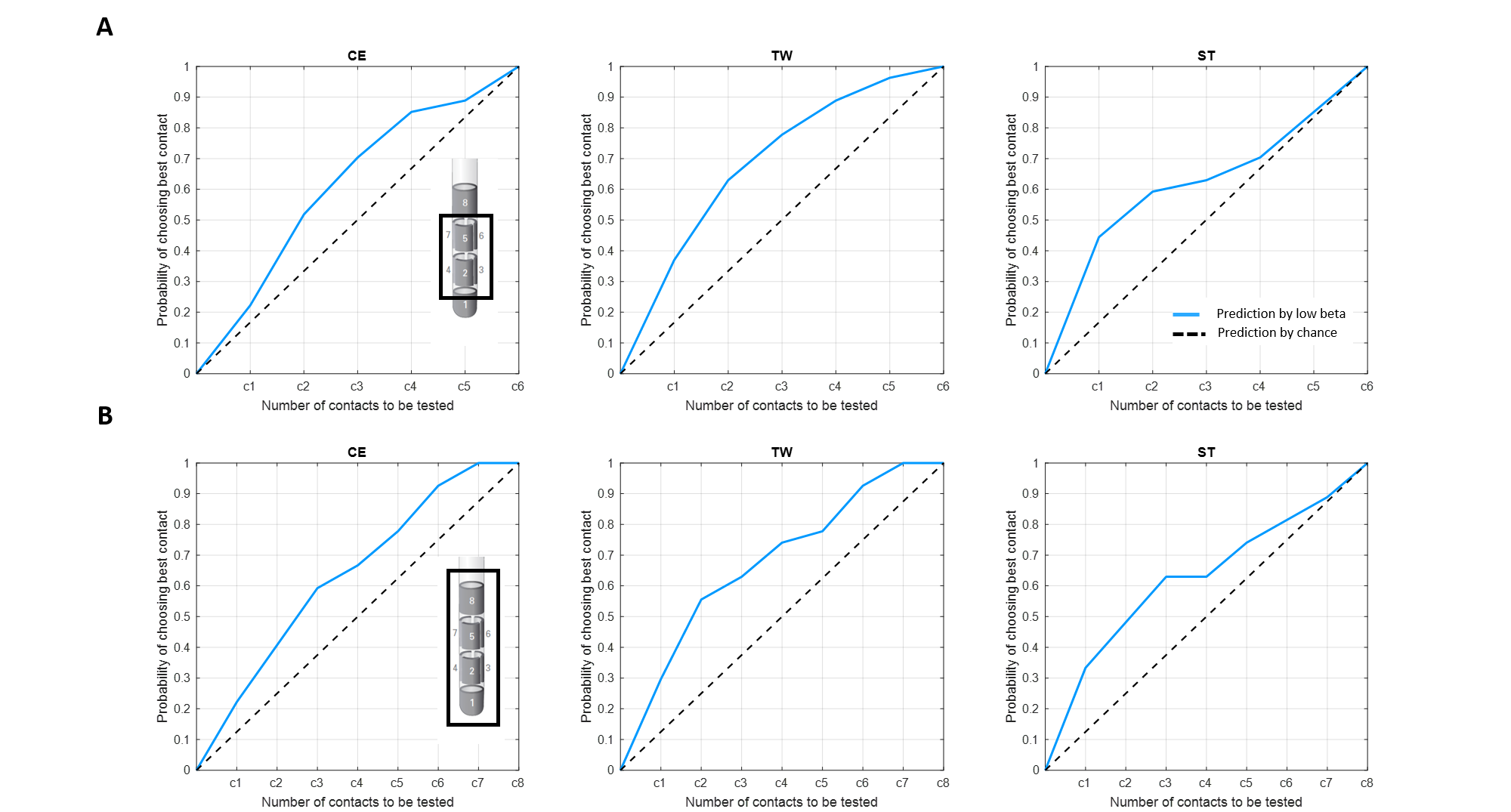


**Supplementary figure 5:** Low beta activity ‐ based contact prediction Illustrates the probability of identifying one of the best stimulation contacts for the three clinical parameters (CE, TW, ST) out of the 6 segmented contacts (A) and out of all 8 contacts (B) across the 27 hemispheres. In contrast to figure 4A and 5A in which the probability of identifying the highest ranked stimulation contact based on selection and combination of multiple features is illustrated, the current figure illustrates the probability of identifying the best stimulation contact solely based on low beta activity. The dashed black lines illustrate the prediction by chance (conventional gold standard test strategy), where the probability of identifying the most efficient stimulation contact increases by 0.17 (in A) and 0.125 (in B) after each contact tested. By using low beta activity for contact prediction strategy, after considering up to half of the electrophysiologically ranked stimulation contacts, the probability of identifying one the best stimulation contacts can reach the values as follows: For segmented contacts (A) CE: 70%, TW: 78% and ST: 63%; for all contacts (B) CE: 67%, TW: 74% and ST: 63%; CE: Clinical efficacy, TW: therapeutic window; ST: Side-effect threshold.


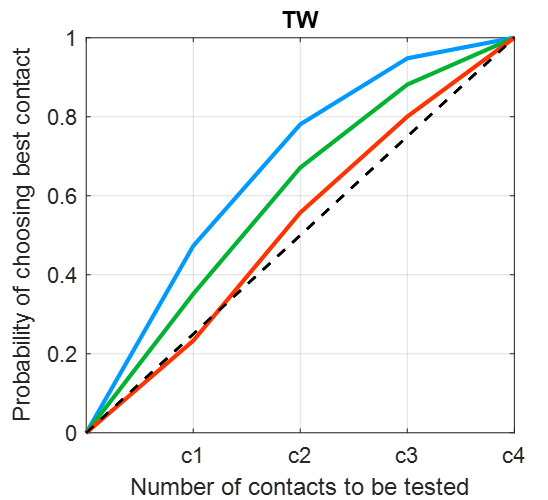


**Supplementary figure 6.** LFP‐based prediction of the chronic stimulation setting. Illustrates the output of the 2nd step of the contact prediction method, corresponding to the the probability of identifying the contact level with the broadest therapeutic window used for chronic stimulation across all 27 hemispheres. Maximum, average and minimum prediction accuracy evaluated on the hold-out set of 9 hemispheres are illustrated as mean and standard deviation (shaded area). The dashed black line illustrates the prediction by chance (conventional gold standard test strategy) (the probability of identifying the most efficient stimulation contact increases by 0.25). The probability of identifying the best stimulation level reaches a maximum of 78.1% when considering 2 out of the 4 levels available.


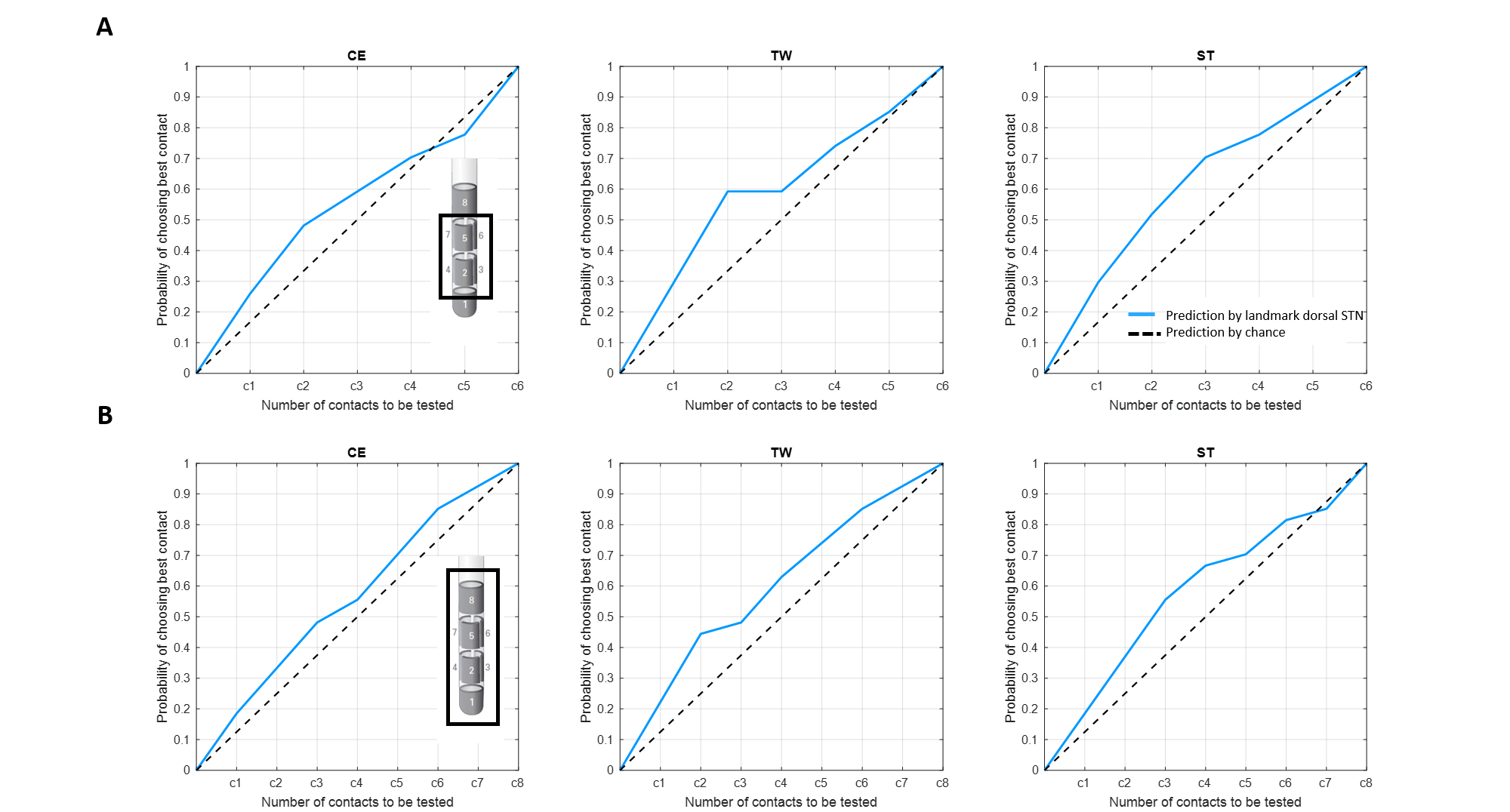


**Supplementary figure 7:** Anatomical (dorsal STN) landmark ‐ based contact prediction Illustrates the probability of identifying one of the best stimulation contacts for the three clinical parameters (CE, TW, ST) out of the 6 segmented contacts (A) and out of all 8 contacts (B) across the 27 hemispheres. In contrast to figure 4A and 5A in which the probability of identifying the highest ranked stimulation contact based on selection and combination of multiple electrophysiological features is illustrated, the current figure illustrates the probability of identifying the best stimulation contact solely based on the distance to an anatomical landmark within the dorsal STN. The dashed black lines illustrate the prediction by chance (conventional gold standard test strategy), where the probability of identifying the most efficient stimulation contact increases by 0.17 (in A) and 0.125 (in B) after each contact tested. By using the anatomical landmark within the dorsal STN for contact prediction strategy, after considering up to half of the ranked stimulation contacts, the probability of identifying one the best stimulation contacts can reach the predictive values as follows: For segmented contacts (A) CE: 60%, TW: 60% and ST: 70%; for all contacts (B) CE: 55%, TW: 63% and ST: 67%; CE: Clinical efficacy, TW: therapeutic window; ST: Side-effect threshold.

**Statistics tables:**

**Effect size legend**

d=0.01--> very small effect size
d=0.20--> small effect size
d=0.50--> medium effect size
d=0.80--> large effect size
d=1.20--> very large effect size
d = 2.00 --> huge effect size

Figure 4B

Clinical efficacy

Friedmann test: x^2^(3) = 36.26, p = <0.001

| Comparison | 1 feat vs 2 feat | 1 feat vs 3 feat | 1 feat vs 4 feat | 1 feat vs 5 feat |
| --- | --- | --- | --- | --- |
| P-values | 0.02 | <0.001 | <0.001 | <0.001 |
| Effect-size | 0.23 | 0.60 | 0.75 | 0.84 |

Therapeutic Window

Friedmann test: x^2^(3) = 36.88, p = <0.001

| Comparison | 1 feat vs 2 feat | 1 feat vs 3 feat | 1 feat vs 4 feat | 1 feat vs 5 feat |
| --- | --- | --- | --- | --- |
| P-values | <0.001 | <0.001 | <0.001 | <0.001 |
| Effect-size | 0.46 | 0.88 | 1.01 | 1.13 |

Side effect Threshold

Friedmann test: x^2^(3) = 28.94, p = <0.001

| Comparison | 1 feat vs 2 feat | 1 feat vs 3 feat | 1 feat vs 4 feat | 1 feat vs 5 feat |
| --- | --- | --- | --- | --- |
| P-values | 0.64 | 0.002 | <0.001 | <0.001 |
| Effect-size | 0.05 | 0.33 | 0.52 | 0.63 |

Figure 5B

Clinical efficacy

Friedmann test: x^2^(3) = 53.94, p = <0.001

| Comparison | 1 feat vs 2 feat | 1 feat vs 3 feat | 1 feat vs 4 feat | 1 feat vs 5 feat |
| --- | --- | --- | --- | --- |
| P-values | 0.003 | <0.001 | <0.001 | <0.001 |
| Effect-size | 0.31 | 0.85 | 1.01 | 1.21 |

Therapeutic Window

Friedmann test: x^2^(3) = 50.25

| Comparison | 1 feat vs 2 feat | 1 feat vs 3 feat | 1 feat vs 4 feat | 1 feat vs 5 feat |
| --- | --- | --- | --- | --- |
| P-values | 0.02 | <0.001 | <0.001 | <0.001 |
| Effect-size | 0.24 | 0.50 | 0.78 | 0.96 |

Side effect Threshold

Friedmann test: x^2^(3) = 5.19, p = 0.159

| Comparison | 1 feat vs 2 feat | 1 feat vs 3 feat | 1 feat vs 4 feat | 1 feat vs 5 feat |
| --- | --- | --- | --- | --- |
| P-values | <0.001 | <0.001 | <0.001 | <0.001 |
| Effect-size | 0.35 | 0.42 | 0.48 | 0.52 |

Figure 7 A

Clinical efficacy

| Comparison | Beta vs Max. Prediction | | Beta vs Mean Prediction | | Beta vs Min. Prediction | |
| --- | --- | --- | --- | --- | --- | --- |
| Contacts | 1/6 | 3/6 | 1/6 | 3/6 | 1/6 | 3/6 |
| P-values | <0.001 | <0.001 | <0.001 | <0.001 | <0.001 | 0.01 |
| Effect-size | 3.77 | 4.10 | 2.35 | 1.65 | 0.50 | 0.26 |

Therapeutic Window

| Comparison | Beta vs Max. Prediction | | Beta vs Mean Prediction | | Beta vs Min. Prediction | |
| --- | --- | --- | --- | --- | --- | --- |
| Contacts | 1/6 | 3/6 | 1/6 | 3/6 | 1/6 | 3/6 |
| P-values | <0.001 | <0.001 | 0.99 | <0.001 | <0.001 | <0.001 |
| Effect-size | 1.83 | 1.83 | 0.01 | 0.64 | 1.92 | 2.91 |

Side effect Threshold

| Comparison | Beta vs Max. Prediction | | Beta vs Mean Prediction | | Beta vs Min. Prediction | |
| --- | --- | --- | --- | --- | --- | --- |
| Contacts | 1/6 | 3/6 | 1/6 | 3/6 | 1/6 | 3/6 |
| P-values | 0.27 | <0.001 | <0.001 | 0.58 | <0.001 | <0.001 |
| Effect-size | 0.12 | 2.30 | 2.11 | 0.06 | 4.16 | 1.91 |

Figure 7B

Clinical efficacy

| Comparison | Beta vs Max. Prediction | | Beta vs Mean Prediction | | Beta vs Min. Prediction | |
| --- | --- | --- | --- | --- | --- | --- |
| Contacts | 1/8 | 4/8 | 1/8 | 4/8 | 1/8 | 4/8 |
| P-values | <0.001 | <0.001 | <0.001 | <0.001 | 0.016 | <0.001 |
| Effect-size | 4.44 | 3.25 | 2.00 | 0.57 | 0.24 | 1.92 |

Therapeutic Window

| Comparison | Beta vs Max. Prediction | | Beta vs Mean Prediction | | Beta vs Min. Prediction | |
| --- | --- | --- | --- | --- | --- | --- |
| Contacts | 1/8 | 4/8 | 1/8 | 4/8 | 1/8 | 4/8 |
| P-values | <0.001 | <0.001 | <0.001 | 0.5977 | <0.001 | <0.001 |
| Effect-size | 2.54 | 2.32 | 0.87 | 0.42 | 1.72 | 3.09 |

Side effect Threshold

| Comparison | Beta vs Max. Prediction | | Beta vs Mean Prediction | | Beta vs Min. Prediction | |
| --- | --- | --- | --- | --- | --- | --- |
| Contacts | 1/8 | 4/8 | 1/8 | 4/8 | 1/8 | 4/8 |
| P-values | <0.001 | <0.001 | <0.001 | 0.13 | <0.001 | <0.001 |
| Effect-size | 1.04 | 2.37 | 0.78 | 0.15 | 2.88 | 2.25 |

Figure 8A

Clinical efficacy

| Comparison | Prediction with/without Anatomy |
| --- | --- |
| P-values | 0.002 |
| Effect-size | 0.32 |

Therapeutic Window

| Comparison | Prediction with/without Anatomy |
| --- | --- |
| P-values | <0.001 |
| Effect-size | 0.53 |

Figure 8B

Clinical efficacy

| Comparison | Prediction with/without Anatomy |
| --- | --- |
| P-values | <0.001 |
| Effect-size | 1.15 |

Therapeutic Window

| Comparison | Prediction with/without Anatomy |
| --- | --- |
| P-values | <0.001 |
| Effect-size | 0.83 |

**References**

1. Horn, A., et al., *Lead-DBS v2: Towards a comprehensive pipeline for deep brain stimulation imaging.* NeuroImage, 2019. **184**: p. 293-316.

2. Levy, J.P., et al., *Structure-function relationship of the posterior subthalamic area with directional deep brain stimulation for essential tremor.* Neuroimage Clin, 2020. **28**: p. 102486.

3. Avants, B.B., et al., *The Insight ToolKit image registration framework.* Front Neuroinform, 2014. **8**: p. 44.

4. Schönecker, T., et al., *Automated optimization of subcortical cerebral MR imaging-atlas coregistration for improved postoperative electrode localization in deep brain stimulation.* AJNR Am J Neuroradiol, 2009. **30**(10): p. 1914-21.

5. Husch, A., et al., *PaCER - A fully automated method for electrode trajectory and contact reconstruction in deep brain stimulation.* Neuroimage Clin, 2018. **17**: p. 80-89.

6. Hellerbach, A., et al., *DiODe: Directional Orientation Detection of Segmented Deep Brain Stimulation Leads: A Sequential Algorithm Based on CT Imaging.* Stereotact Funct Neurosurg, 2018. **96**(5): p. 335-341.

7. Ewert, S., et al., *Toward defining deep brain stimulation targets in MNI space: A subcortical atlas based on multimodal MRI, histology and structural connectivity.* NeuroImage, 2018. **170**: p. 271-282.

8. Nguyen, T.A.K., et al., *Analysis of patient-specific stimulation with segmented leads in the subthalamic nucleus.* PLoS One, 2019. **14**(6): p. e0217985.

9. Tibshirani, R., *Regression Shrinkage and Selection via the Lasso.* Journal of the Royal Statistical Society. Series B (Methodological), 1996. **58**(1): p. 267-288.

10. Tinkhauser, G., et al., *Directional local field potentials: A tool to optimize deep brain stimulation.* Mov Disord, 2018. **33**(1): p. 159-164.
